# Extreme Heat Exposure and Dementia-Related Mortality: A Systematic Review and Meta-Analysis

**DOI:** 10.3390/jcm15218655

**Published:** 2026-11-06

**Authors:** Jung-Eun Lee, Gyu Tae Lee, Han-A Cho

**Affiliations:** 1 Department of Counseling Psychology, Shinhan University, Uijeongbu 11644, Gyeonggi-do, Republic of Korea; jelee@shinhan.ac.kr; 2 Department of Big Data Management, Shinhan University, Uijeongbu 11644, Gyeonggi-do, Republic of Korea; lgstar728@naver.com; 3 Department of Dental Hygiene, Shinhan University, Uijeongbu 11644, Gyeonggi-do, Republic of Korea

**Keywords:** dementia, extreme heat, heatwave, mortality, older adults, Alzheimer’s disease, climate change, systematic review, meta-analysis

## Abstract

**Background/Objectives**: The world is undergoing rapid population aging while experiencing more frequent and intense heatwaves driven by climate change. People living with dementia—who can have impaired thermoregulation, a reduced ability to perceive and respond to heat, and high care dependency—may be especially susceptible to heat, but the evidence on mortality has not been synthesized. We conducted a systematic review and meta-analysis to quantify the association between heatwave exposure and dementia-related mortality. **Methods**: PubMed, Web of Science Core Collection, ProQuest Dissertations & Theses Global, and APA PsycINFO were searched from inception to 15 June 2026; the searches were rerun on 15 October 2026 and supplemented by citation searching and by a PubMed search without the older-adult concept block. The review is reported according to PRISMA 2020 (PROSPERO CRD420261436457, registered after the searches had been run). Observational studies reporting relative effect estimates for mortality during defined heatwave or extreme heat events, among people with diagnosed dementia or for deaths attributed to dementia in mortality records, were eligible. Risk of bias was assessed with an adapted ROBINS-E approach and the certainty of evidence with GRADE. Odds ratios (ORs) from time-stratified case-crossover analyses, which estimate rate ratios, were pooled in a random-effects model with restricted maximum-likelihood estimation and Hartung–Knapp 95% confidence intervals (CIs); heterogeneity was described with Cochran’s *Q*, *I*^2^, and a 95% prediction interval. The primary analysis was restricted to studies with a dementia-specific outcome definition and an estimate of the total heatwave association and used one study from each pair of studies based on the same mortality data. **Results**: Six studies conducted in China and Australia met the eligibility criteria, one of them identified only by the supplementary search; three contributed to the primary analysis, and three were used in sensitivity analyses only. All study-level estimates exceeded unity, but the pooled association was imprecise and not statistically significant (OR 1.84, 95% CI 0.54–6.25), with high heterogeneity (*I*^2^ = 94.9%; 95% prediction interval 0.21–16.31). In sensitivity analyses that retained at least three studies, pooled ORs ranged from 1.50 to 1.84; analyses reduced to two studies had uninformative CIs. The CI excluded the null when an alternative national study was substituted (OR 1.76, 95% CI 1.13–2.72), when all six estimates were pooled without regard to overlapping data (OR 1.57, 95% CI 1.15–2.15), or when the conventional DerSimonian–Laird method was used. No included study compared dementia with non-dementia deaths, and only one was restricted to adults aged 60 years or older; the others were predominantly older or did not report age. The certainty of evidence was very low. **Conclusions**: The evidence is inconclusive regarding the magnitude and consistency of an association between heatwaves and dementia-related mortality. The study-level estimates point in the same direction, but the pooled estimate is an average across highly heterogeneous settings, is too imprecise to establish an effect, and cannot show whether the relative increase exceeds that for other deaths; evidence specific to older adults with dementia is largely indirect. Including people with dementia in heat–health action plans remains a reasonable precaution, and independent, methodologically harmonized primary studies are needed.

## 1. Introduction

Anthropogenic climate change has increased the frequency, intensity, and duration of heatwaves across virtually every inhabited region, and these trends are projected to continue throughout the twenty-first century [1,2]. Non-optimal temperatures are a major environmental contributor to preventable mortality, accounting for an estimated several million excess deaths annually between 2000 and 2019, with the heat-attributable share increasing over time [3]. Older adults are consistently identified as particularly susceptible to heat-related mortality because aging reduces thermoregulatory capacity and cardiovascular reserve and frequently co-occurs with chronic disease and polypharmacy [4,5]. Catastrophic heat episodes, such as those reported in Madrid, Italy, and the UK, have produced sharp increases in excess mortality that is concentrated among the oldest and most dependent individuals [6,7,8].

Among the older population, people living with dementia may face particular vulnerabilities to heat that differ from those of other older adults living in the community; with their growing numbers, they are a group for whom tailored support during hot weather warrants attention. The number of adults living with dementia worldwide is projected to rise from an estimated 57 million in 2019 to 153 million by 2050, driven largely by population aging [9,10]. Dementia can affect both the ability to perceive heat stress caused by high temperatures and the capacity to respond to it, for example by maintaining hydration or using air conditioning: neurodegenerative changes may affect hypothalamic thermoregulatory control, autonomic function, and thirst perception, and cognitive and behavioral changes can make it harder for some people to recognize heat, adjust clothing, maintain hydration, or relocate to a cooler environment [11,12]. Body temperature has been associated with cognitive performance in older adults, while altered central and peripheral thermal processing has been documented in Alzheimer’s disease [13,14]. Many people with dementia also receive support from caregivers or live in residential care, and some are prescribed psychotropic or anticholinergic medications that can reduce heat dissipation [15]. These vulnerabilities are not uniform: the experience of dementia varies widely with its type and stage, with co-existing conditions, and with the living and care environment, and many people living with dementia retain the ability to take protective action when given appropriate information and support. Population-level risk estimates should therefore inform, rather than replace, an understanding of each person’s specific circumstances.

A growing body of epidemiological research has examined how extreme heat affects people with dementia and related conditions, but the evidence is fragmented across heterogeneous outcomes. Several studies have linked high ambient temperature to increased hospital admissions or emergency care for dementia and Alzheimer’s disease [16,17,18,19,20,21,22], and a parallel literature reports adverse effects of heat on cognition and cognitive decline in older populations [23,24,25,26,27,28,29,30]. Others have investigated thermal comfort and physiological responses in residential and aged-care settings [31,32], examined heat in relation to the onset of dementia [33], or projected future heat-related dementia burdens under climate scenarios [19,34]. Evidence on mortality, by contrast, remains comparatively sparse and dispersed; heat-related outcomes in dementia have most often been reported within broader analyses of mental and behavioral disorders [35,36,37,38,39,40], among heat-vulnerable subgroups [41,42,43], or in relation to atypical clinical presentation [44], and only a few studies have addressed mortality among people with dementia directly [45,46]. Individual studies are further constrained by small numbers of dementia deaths, divergent heatwave definitions, and inconsistent age criteria. A recent systematic review of ambient temperature and mental and neurological conditions in adults aged 60 years or older pooled heatwave-related mortality for mental disorders as a broad category (RR 1.27, 95% CI 0.96–1.68; four studies) [47]; to our knowledge, no quantitative synthesis has focused on heatwave-related dementia mortality.

To address this gap, we conducted a systematic review and meta-analysis to quantify the association between heatwave exposure and dementia-related mortality. In line with the registered review question, our a priori hypothesis was that heatwave exposure is associated with higher mortality. No direction was hypothesized for differences by country, heatwave temperature threshold, or heatwave duration, and these comparisons were treated as exploratory. We pooled relative effect estimates using a random-effects model, quantified between-study heterogeneity, assessed the certainty of the evidence, and described variation by country and heatwave definition. By synthesizing the available evidence, we sought to clarify the magnitude and consistency of the association and to inform heat–health adaptation and clinical care. Throughout, we use “heatwave” for the exposure contrast analyzed (a discrete event defined by an explicit temperature threshold, usually with a minimum duration) and “extreme heat” for the broader hazard.

## 2. Materials and Methods

### 2.1. Protocol and Reporting

The review is reported following the PRISMA 2020 guideline [48], and the completed checklist is given in Supplementary Table S1. It was registered in PROSPERO (Centre for Reviews and Dissemination, University of York, York, UK; CRD420261436457) on 12 August 2026. The registration was not prospective: the database searches had been run on 15 June 2026, and record screening was under way when the record was submitted. The registered record therefore documents the review plan as it stood at that point and cannot be regarded as a protocol fixed before the candidate studies were known. According to the record, the full protocol uploaded to PROSPERO will be made available after completion of the review; the registered record is therefore the publicly accessible statement of the planned methods, and departures from it are reported below. A related review registered independently by another group (CRD420261356070) addresses the risk of hospitalization and mortality associated with climate-related extreme weather events in general among people living with dementia; the present review differs in focusing on mortality during defined heatwaves or episodes of extreme heat, in targeting older adults, and in pooling relative estimates for heatwave compared with non-heatwave periods.

The review question was framed in terms of population, exposure, comparator, outcome, and study design. As registered, the primary objective was to estimate how heatwave exposure relates to mortality in older adults with a dementia diagnosis; the registered question was directional (whether exposure increases mortality risk) and constitutes the a priori hypothesis of the review. The secondary objective was to explore whether the association differed by country, heatwave temperature threshold, and heatwave duration, for which no direction was hypothesized. As described below, the evidence identified did not correspond fully to the registered question, and the estimand was therefore re-specified during revision as the relative change in dementia-related mortality during heatwave periods compared with non-heatwave periods—that is, mortality among people with a recorded diagnosis of dementia or, in most studies, deaths attributed to dementia in mortality records.

Departures from the registered record are listed in Supplementary Table S7 and fall into four groups. (i) *Age.* An early working plan, which was not registered, defined older adults as those aged 65 years or older. By the time of registration the criterion had been set at 60 years, the cut-off used in a potentially eligible study [49], and the record allowed studies that were not restricted by age to be retained as age-indirect evidence. Both features were adopted after potentially eligible studies had been identified and with knowledge of their populations; they are therefore post hoc decisions even though they appear in the registered record. (ii) *Planned but not carried out.* Study authors were not contacted for missing or unreported data; the search for unpublished studies was limited to one dissertation database; and subgroup analyses by country, temperature threshold, and heatwave duration were replaced by a descriptive stratification because too few studies were available (Section 2.7). (iii) *Carried out but not planned.* The classification of age directness and the related sensitivity analyses were not registered, and neither was a trim-and-fill procedure that was run in the initial analysis; the latter has been withdrawn (Section 3.5). In addition, titles were screened within each database export before de-duplication, whereas the record planned de-duplication before screening (Section 2.4). (iv) *Changed during peer review.* A study that had been included in the initial submission was excluded from the review because its dementia mortality estimate applied only to people aged 15–64 years and therefore provided no evidence on older adults [50]; the primary analysis set was re-specified, first by setting aside one of two studies based on the same national mortality data and then, in the second round of review, by restricting the analysis to estimates with a dementia-specific outcome definition and an estimate of the total heatwave association; the rule for choosing among multiple estimates was formalized; the random-effects estimator was replaced; the risk-of-bias ratings were re-assessed; a rating of the certainty of evidence was added, although the record stated that certainty would not be assessed; the searches were rerun with harmonized strategies and supplemented by citation searching and, in the second round of review, by a PubMed search without the older-adult block, which identified one further eligible study [51]; and the age criterion was reformulated (Section 2.2, Section 2.3, Section 2.5, Section 2.6, Section 2.7 and Section 2.8). The changes in group (iv) were made with knowledge of the initial pooled results; for transparency, the analysis as first conducted (the initially selected estimates pooled with the DerSimonian–Laird estimator) is retained as a sensitivity analysis for the five studies of the initial submission that remain eligible, and the primary analysis of the first revision is also reported as a sensitivity analysis.

### 2.2. Eligibility Criteria

Studies had to meet each of the criteria set out below. *Population.* The target population was older adults (aged 60 years or older) with dementia. Any clearly described method of identifying dementia was accepted, including clinical diagnostic criteria, medical records, hospital or administrative diagnosis codes, disease registries, and death certification. The registered criteria named Alzheimer’s disease, vascular dementia, other specified dementias, and unspecified dementia; the record did not list diagnostic codes, and these categories correspond to codes F00–F03 and G30 of the International Classification of Diseases, 10th Revision (ICD-10). The definitions used in the included studies did not all match these codes, and the criteria as applied were therefore broader than those registered: three studies also counted G31 (other degenerative diseases of the nervous system, which covers frontotemporal and Lewy body dementia) [49,52,53], one study labeled the whole block of F00–F09 (organic, including symptomatic, mental disorders, which also contains delirium and other non-dementia diagnoses) as dementia [54], and one study reported dementia as the sum of Alzheimer’s disease and non-Alzheimer dementias, with the codes listed only in the supplementary material of the original article [51]. These studies were retained with the definitions specified by their authors; for the F00–F09 study, this departs from the registered exclusion of studies in which dementia could not be separated from broader neurological or psychiatric conditions (Supplementary Table S7). Because the F00–F09 block cannot isolate dementia, that estimate was not included in the primary analysis; it is presented as indirect evidence and used only in sensitivity analyses (Section 2.7). Only one study used a definition confined to the registered diagnoses (Alzheimer’s disease, without the codes being reported) [55]; one further study reported an Alzheimer’s disease-specific estimate in addition to its estimate for all dementias [51], and the two Alzheimer’s disease-specific estimates were combined in a sensitivity analysis (Section 2.7). Because population-based mortality studies were rarely restricted by age, the absence of an age restriction was not used to exclude studies; instead, each study was assigned to one of three categories of age directness: (i) restricted to adults aged 60 years or older [49]; (ii) not restricted by age but documented to be predominantly older (mean age above 80 years, more than 80% of deaths at 65 years or older, or most dementia deaths after the age of 80 years) [51,52,53,55]; and (iii) not restricted by age, with the age distribution of dementia deaths not reported [54]. The age criterion as applied was therefore defined by the age range to which an estimate referred: an estimate was eligible if adults aged 60 years or older could be expected to account for most of the dementia deaths on which it was based, that is, if it referred to adults aged 60 years or older, or to all ages or all adults with dementia deaths predominantly at older ages or with the age distribution not reported (categories (i)–(iii)). Estimates confined to younger age bands that include only a small segment of the target population—for example, 15–64 years, in which deaths at 60–64 years cannot be isolated—were not eligible, because they do not represent the target population. Studies whose age range overlapped the target population only in part, such as adults aged 18 years or older, were therefore retained and classified by age directness, and age-specific estimates for older age groups were extracted where reported (Supplementary Table S8). This criterion was made explicit during peer review and reformulated in the second round (Supplementary Table S7). Studies in which cognitive impairment was identified only with screening instruments such as the Mini-Mental State Examination, without a dementia diagnosis, were not eligible.

*Exposure.* Eligible exposures were heatwaves or episodes of extreme heat identified by an explicit temperature threshold, either absolute or percentile-based, with or without a minimum number of consecutive days. Studies of the summer season as a whole, without discrete heat events, were not eligible. Estimates expressed as the change in mortality per 1 °C increase in temperature were also excluded, since they describe a continuous temperature–response relationship and not the effect of a defined heatwave. *Comparator.* Heatwave periods were compared with non-heatwave days or with a study-specific reference exposure. In time-stratified case-crossover studies, the control days came from the same calendar stratum as the case day (usually the same year, month, and day of the week); for continuous or intensity-based heat metrics, the reference was the exposure value with the lowest mortality risk. One study separated the heatwave association into an intensity-related effect, estimated from the temperature–mortality function, and an added effect of heatwave days conditional on temperature [54]; because the added effect is conditional on temperature and therefore does not estimate the total heatwave association estimated by the other studies, neither component was included in the primary analysis, and both were used in sensitivity analyses (Section 2.7).

*Outcome.* The outcome was dementia-related mortality during or after heatwave exposure. Two definitions were accepted: (a) deaths attributed to dementia in mortality records (as the underlying cause of death where this was stated) and (b) deaths occurring in a defined cohort of people with a previous clinical diagnosis of dementia. These definitions do not identify the same epidemiological quantity: the first describes how often dementia-coded deaths occur in the population during heatwaves, whereas the second describes the risk of dying among people living with dementia. Both were eligible, but the outcome type was recorded for every study, and the single cohort-based estimate—deaths with Alzheimer’s disease as the underlying cause within two months of discharge among patients hospitalized for Alzheimer’s disease [55]—was included in the primary analysis for the reasons given in Section 2.7 and removed in a sensitivity analysis. A study had to report a relative effect estimate together with a measure of its uncertainty, or data from which both could be derived. Mortality estimates for the general population in which a dementia group could not be identified separately were not eligible, and studies restricted to hospitalization, emergency department use, morbidity, cognitive decline, or physiological responses were outside the scope of the quantitative synthesis. *Study design and publication characteristics.* Original observational research with any longitudinal exposure design (cohort, case-crossover, time-series, or ecological) was eligible, without restriction on country or year of publication; only reports published in English were considered. Secondary or non-empirical publications (reviews, protocols, editorials, and commentaries), conference abstracts, case reports, qualitative research, and animal experiments were not eligible.

### 2.3. Information Sources and Search Strategy

The original search covered four sources from their inception to 15 June 2026: PubMed, Web of Science Core Collection, ProQuest Dissertations & Theses Global, and APA PsycINFO (via EBSCOhost). PubMed was chosen for biomedical and public-health research, Web of Science Core Collection for multidisciplinary coverage that includes the environmental sciences, APA PsycINFO for behavioral and mental-health research on dementia, and ProQuest Dissertations & Theses Global as a source of unpublished theses. Three concept blocks (dementia, heatwaves or extreme heat, and older adults) were joined with Boolean operators, using subject headings and free-text terms suited to each database, and retrieval was limited to English-language records. Only the PubMed strategy had been saved verbatim at the time of the original search; the strategies for the other three databases were reconstructed afterwards from the search record and were not fully harmonized across databases (Supplementary Table S2, Part A). To make the search reproducible, the searches were rerun on 15 October 2026, from database inception and limited to English-language records, with harmonized strategies that are reported in full in Supplementary Table S2, Part B. PubMed, Web of Science Core Collection, and ProQuest Dissertations & Theses Global were searched again, the last with two variants of the strategy in which either the dementia block or the older-adult block was searched without the restriction to fields other than the full text that was applied to the remaining blocks; in place of the single-database search of APA PsycINFO, the EBSCOhost Complete Package was searched in subject headings, titles, and abstracts. On the same date, citation searching was carried out: the reference lists of the five studies then included [49,52,53,54,55] and of a related review [47] were screened, and records citing the included studies were identified in Web of Science (Supplementary Table S2, Part B). Because the review addresses dementia-related mortality, and most included studies were not restricted by age, the older-adult block could have excluded eligible studies that did not refer to age. In the second round of review, a supplementary PubMed search was therefore run on 22 October 2026 with the dementia and heat blocks of the harmonized PubMed strategy and the English-language limit, restricted to records entered in PubMed up to 15 October 2026, and with the records retrieved by the older-adult block removed (Boolean NOT) so that it returned only records that the older-adult block had excluded; because of the length limit of the search interface, it was run as four sub-queries whose union is equivalent to the full query (Supplementary Table S2, Part C). PubMed was chosen because all included studies are indexed in it. Generative artificial intelligence was used for exploratory background reading before the formal search and, in the second round of review, to run the supplementary PubMed search through a PubMed search interface and to pre-screen its records (Section 2.4; Acknowledgments); it was not itself an information source, and every record that was screened, assessed, or included was retrieved from the bibliographic databases or by citation searching. Embase and Scopus were not searched, and the databases other than PubMed were not searched without the older-adult block, which is acknowledged as a limitation (Section 4.5).

### 2.4. Selection Process

The records retrieved from each database were exported to Microsoft Excel (Microsoft Corporation, Redmond, WA, USA). Selection followed the sequence below, which is reported as it was actually carried out. First, within each database export, titles were screened to remove records that were clearly unrelated to the review question. Second, the records retained from the four databases were merged, and duplicates were identified in Excel and verified manually against the title, authors, publication year, and other bibliographic details. Third, the titles and abstracts of the de-duplicated records were assessed against the eligibility criteria; a record judged potentially eligible by at least one reviewer was carried forward to the next stage. Fourth, the full-text reports were assessed, and a single primary reason for exclusion was recorded for each ineligible report (Supplementary Table S6). At every stage, two reviewers worked independently; disagreements were settled by discussion and, where consensus was not reached, by a third reviewer. Because de-duplication followed the title-level screening, this order differs from the PRISMA 2020 recommendation to remove duplicates before screening; consequently, the number of unique records among those initially retrieved is not available, and the flow diagram gives the counts at each stage as they occurred. The records retrieved in the rerun were handled in the same way: the exports from the four sources were merged in Microsoft Excel; duplicates within the rerun and records already retrieved in the original search were identified by DOI and by title, authors, and publication year and removed; the remaining records were screened on title and abstract; and potentially eligible records were assessed in full text by two reviewers working independently and against the same eligibility criteria. The number of records removed or excluded at each of these steps was not documented for the rerun, so only the number retrieved from each source is reported (Supplementary Table S2, Part B), and the rerun is shown separately in the flow diagram. Reports identified by citation searching were assessed in full text. The records retrieved by the supplementary PubMed search were first restricted to those mentioning mortality or death in any searchable field, because a record without these terms cannot report a mortality estimate; the remaining records were screened on title and abstract, potentially eligible reports were assessed in full text, and the number of records at each step and the reason for each full-text exclusion are reported (Section 3.1; Supplementary Tables S2, Part C, and S6). For this search only, the records were pre-screened against the eligibility criteria with the assistance of generative AI (Acknowledgments), and every screening and full-text decision was verified by the authors. Reports that analyzed the same deaths as an included study were treated as additional reports of that study.

### 2.5. Data Extraction

Data were extracted with a structured form that was prepared in advance and piloted on a subset of the eligible studies. Extraction was done independently by two reviewers; discrepancies were resolved by returning to the original article, with a third reviewer consulted when needed. The items recorded were publication details, country and study period, mortality data source, age and other participant characteristics, method of dementia ascertainment, study design and statistical model, heatwave definition and exposure assessment, lag period, adjustment variables, mortality outcome, the original effect measure, and each effect estimate with its confidence interval. Study authors were not contacted for unreported data. To identify overlap, the country and region, study years, mortality data source, and age range of every included study were mapped (Supplementary Table S4). When two studies drew on the same mortality data source for overlapping areas and years, the study with the longer study period, the larger number of deaths, and the population most directly matching the target population was included in the primary analysis, and the other study was used only in sensitivity analyses. Several studies reported estimates for multiple heat thresholds, durations, lag periods, and heat metrics. Selection among them was governed by an explicit hierarchy, which was formalized post hoc during peer review and applied identically to every study: (1) the estimate for all dementia deaths in the whole study population rather than for a subgroup; (2) a percentile-based definition with the threshold closest to the 95th percentile (the threshold common to most studies), or the absolute threshold when no percentile-based definition was reported; (3) a minimum duration of three consecutive days when an estimate was reported numerically for that duration, and otherwise two days; (4) a definition based on daily mean temperature, or the daytime metric when only daytime and nighttime metrics were reported; (5) the cumulative lag period and the main model designated by the original authors, rather than sensitivity models; and (6) estimates given as numbers in the text, tables, or figure labels rather than read from plotted points. The rules were applied in this order among the dementia-specific estimates that were reported numerically. The estimate selected for each study, its location in the original publication, and the rules that determined the selection are shown in Supplementary Table S5, and the numerically reported estimates for alternative definitions are listed in Supplementary Table S8. For four studies, the hierarchy selected the same estimate as had been used before it was formalized; one of these studies examined three percentile thresholds but reported a numerical estimate only for the 95th percentile [55]. For the remaining study [49], the 90th-percentile, two-day estimate had been used initially; under the hierarchy, the estimate for the 95th percentile and three or more days, given in the same publication, was used, and the 90th-percentile and the 95th-percentile two-day estimates were examined in sensitivity analyses. For the study identified in the second round of review [51], only the definition selected by the original authors from 15 candidate definitions (92.5th percentile for at least four days) was reported numerically, so rule 6 determined the selection; its estimate for Alzheimer’s disease deaths was extracted for a sensitivity analysis.

### 2.6. Risk-of-Bias Assessment

Risk of bias was appraised with an adapted version of ROBINS-E, the Risk Of Bias In Non-randomized Studies of Exposures instrument [56], which suits observational studies of an environmental exposure. The assessment used the seven ROBINS-E domains and the tool’s judgment categories (low risk, some concerns, high risk, and very high risk of bias), but it was adapted in one important respect: domain-level judgments were reached with review-specific criteria that operationalize each domain for studies of heat and dementia mortality (Supplementary Table S3), rather than by answering the full set of ROBINS-E signaling questions and applying the domain algorithms. We therefore describe it throughout as an adapted ROBINS-E assessment; its judgments are not equivalent to those of a complete assessment with the instrument (Section 4.5). The overall judgment for each study was derived from the domain-level judgments without a numerical score, following the principle that the overall risk of bias is at least as severe as the most severe domain-level judgment: low risk when all domains were at low risk; some concerns when at least one domain raised some concerns and none was at high or very high risk; high risk when at least one domain was at high risk; and very high risk when at least one domain was at very high risk or several domains were at high risk. Two reviewers rated the studies independently after calibrating their judgments on one eligible study, and differences were resolved by consensus or by a third reviewer. During revision, every rating was re-assessed against the written criteria, because several of the initial ratings were more favorable than the criteria allowed; the revised ratings and the rationale for each are given in Supplementary Table S3. The ratings were used in grading the certainty of evidence (Section 2.8) and in interpreting the pooled estimate, and not only as a descriptive summary.

### 2.7. Statistical Analysis

All analyses were run in R (version 4.3.3; R Foundation for Statistical Computing, Vienna, Austria) with the metafor package (version 4.4-0) [57]; the code and the study-level data are provided as Supplementary Files S1 and S2. The initial analysis, which is retained as a sensitivity analysis, had been carried out in Comprehensive Meta-Analysis version 4 (Biostat, Englewood, NJ, USA) [58]. *Effect measure.* All six included studies reported odds ratios (ORs) estimated by conditional logistic regression in time-stratified case-crossover analyses, in one study within a cohort of patients discharged after hospitalization [55]. In a time-stratified case-crossover design, the conditional OR estimates the rate ratio for a transient exposure [59]. The ORs were therefore pooled as reported, on the natural-logarithm scale, and the pooled OR is interpreted as the relative change in the dementia-related mortality rate during heatwave periods compared with non-heatwave periods. The ORs were not converted to risk ratios, because conversion formulas require the risk of the outcome in the unexposed group, which is not defined in a case-crossover design. The standard error of each log OR was derived from the reported 95% confidence interval (CI) as (ln upper limit − ln lower limit)/(2 × 1.96). *Model.* Because the studies differed in region, heatwave definition, design, population, and exposure assessment, a random-effects model was used. The between-study variance (*τ*^2^) was estimated by restricted maximum likelihood (REML), and the CI of the pooled estimate was calculated using the Hartung–Knapp adjustment, which is based on the *t* distribution and performs better than the conventional approach when few studies are available and heterogeneity is high [60]. The approach used in the initial analysis—the DerSimonian–Laird estimator with a normal-approximation (Wald-type) CI [61]—was retained as a sensitivity analysis. Two-sided *p* values below 0.05 were regarded as statistically significant. Heterogeneity was examined with Cochran’s *Q* test and quantified with *I*^2^, for which values below 25%, between 25% and 75%, and above 75% were taken to represent low, moderate, and high heterogeneity [62]. The likely spread of true effects across settings was expressed as a 95% prediction interval [63].

*Primary and sensitivity analyses.* In response to peer review, the primary analysis was restricted to estimates that share a common estimand, defined by two conditions: (1) a dementia-specific outcome definition—dementia or Alzheimer’s disease codes, excluding broader diagnostic blocks that also contain non-dementia conditions—and (2) an estimate of the total heatwave association, that is, the contrast between heatwave and non-heatwave days that is not conditional on temperature. Of each pair of studies based on the same mortality data source, one was set aside according to the rule in Section 2.5 [52,53]. Three studies met these conditions [49,51,55]. The study that used the block F00–F09 and reported the heatwave effect conditional on temperature [54] met neither condition and is presented as indirect evidence. The three studies in the primary analysis differ in the population in which deaths were observed: two analyzed dementia-coded deaths in population mortality records [49,51], and one analyzed deaths from Alzheimer’s disease after discharge in a cohort hospitalized for Alzheimer’s disease [55]. They were combined because all three estimate, within the same self-matched time-stratified case-crossover framework, the rate ratio of dementia-related death on heatwave compared with non-heatwave days for the same type of exposure contrast, and because the post-discharge cohort is the only study that followed people with a recorded dementia diagnosis—the population named in the registered review question—so excluding it would remove the most direct evidence on that population. Because the two outcome types do not identify the same epidemiological quantity, the pooled estimate is interpreted as an average across them, and a restricted synthesis of the population-based studies is reported alongside it. Sensitivity analyses examined (a) population overlap and residual dependence by substituting the alternative study from each overlapping pair [52,53], by excluding the national dataset, and by refitting the primary model as a multivariate random-effects model (REML with a *t*-based CI) with assumed correlations of 0 to 0.8 between the estimates of the national [49] and provincial [51] datasets—even if every dementia death in the provincial dataset were also counted in the national dataset, the shared deaths would make up at most 16.1% of the national deaths (64,226 of 399,036), which implies a correlation of no more than about 0.4 (the square root of the shared fraction); (b) outcome definition and estimand by restricting the synthesis to population-based dementia-coded mortality [49,51] or to Alzheimer’s disease-specific estimates [51,55], and by adding the indirect evidence from [54] with its added effect or its intensity-related effect; (c) age by restricting the evidence to the study of adults aged 60 years or older [49] and by omitting the provincial study of all adult ages [51]; (d) estimate selection by using the alternative estimates described in Section 2.2 and Section 2.5; and (e) the statistical method, including pooling of all eligible estimates and the analysis as first conducted. The primary analysis of the first revision (four studies, including [54]) is also reported. Each study in the primary analysis was omitted in turn (leave-one-out analysis); because this leaves two studies, the resulting Hartung–Knapp CIs, which are based on a *t* distribution with one degree of freedom, are reported for completeness but are not informative, and the same applies to the other analyses of two studies. Leave-one-out analysis assesses the influence of individual studies under the same modeling assumptions and was not interpreted as evidence of robustness to the definition of the effect measure, population eligibility, overlapping datasets, or the choice of estimator. *Exploratory stratification.* The registered record listed subgroup analyses by country, heatwave temperature threshold, and heatwave duration. Because most categories contained only one or two studies and study characteristics were confounded with one another, formal tests of subgroup differences were not performed; study-level estimates are instead presented descriptively by category, and a single study was not treated as a pooled subgroup. Estimates reported within the same study for different thresholds and durations were tabulated (Supplementary Table S8); they were not combined in a multilevel model, because the covariances among estimates derived from the same deaths were not reported and only three studies provided numerical estimates for more than one definition. No included study reported an estimate for non-dementia or all-cause mortality under the same exposure contrast, so a ratio of relative effects comparing dementia with other deaths could not be calculated. Study-specific and pooled estimates are displayed in a forest plot. Meta-regression had been planned only if at least 10 independent studies were available for a candidate moderator and was therefore not performed. *Small-study effects.* With fewer than 10 studies and high heterogeneity, neither a funnel plot nor a formal test such as Egger’s regression can separate small-study effects from other sources of asymmetry [64]; a funnel plot is provided for completeness in Supplementary Figure S1, and no inference about publication bias was drawn from it.

### 2.8. Certainty Assessment

The certainty of the body of evidence for the association between heatwave exposure and dementia-related mortality was rated using the Grading of Recommendations Assessment, Development and Evaluation (GRADE) approach [65]. Evidence from observational studies started at low certainty and could be rated down for risk of bias (informed by the adapted ROBINS-E judgments), inconsistency, indirectness, imprecision, and publication bias, and rated up for a large magnitude of effect, an exposure–response gradient, or plausible residual confounding that would oppose the observed association. The ratings were agreed by consensus among the authors.

## 3. Results

### 3.1. Study Selection

**Figure 1:**
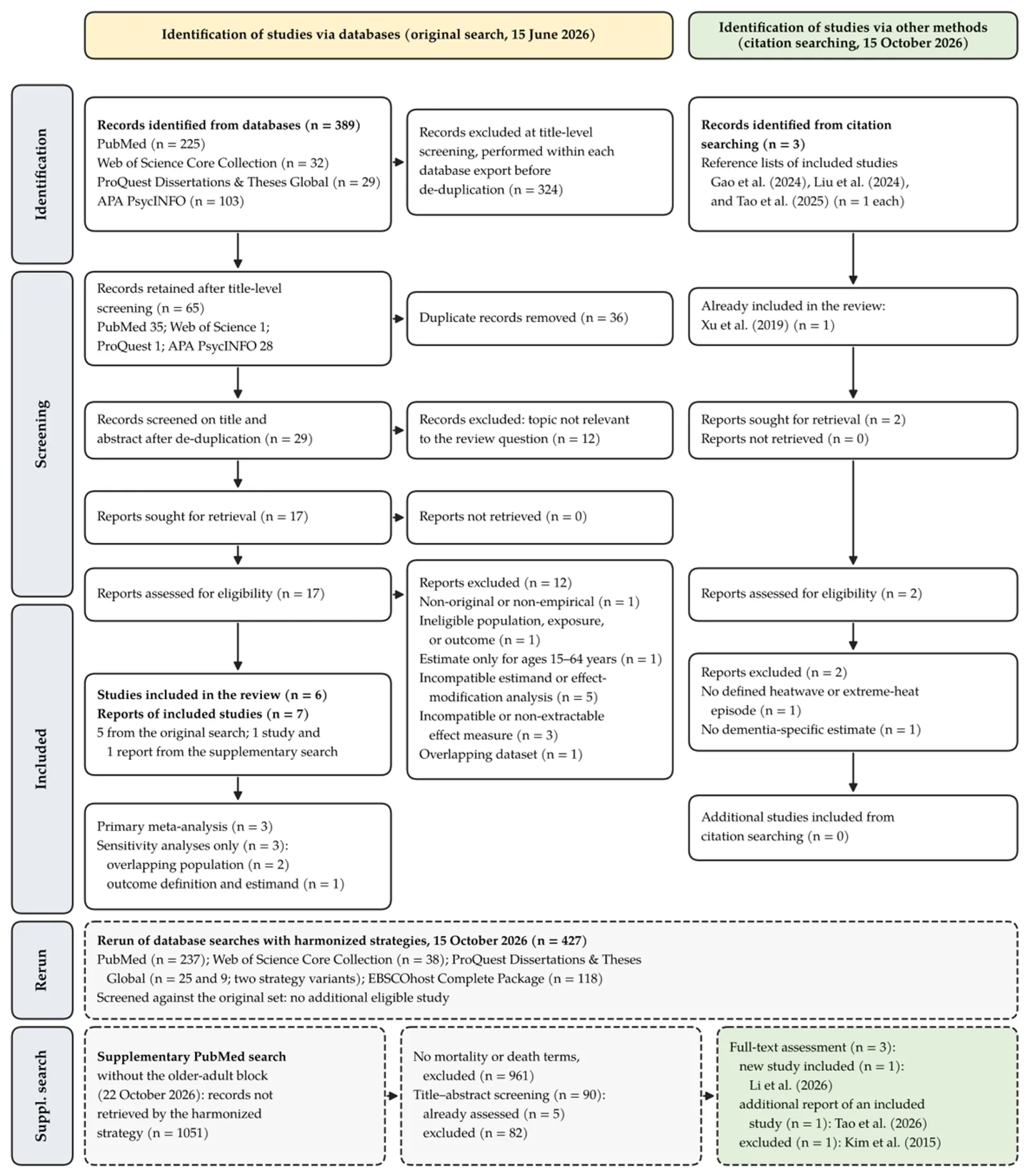
Flow of records through the review, in PRISMA 2020 format. Of 389 records identified in four databases in the original search, 324 were excluded at title-level screening performed within each database export and 36 were removed as duplicates; 29 records were screened on title and abstract, 12 were excluded, and 17 full-text reports were retrieved and assessed for eligibility. Twelve reports were excluded for the reasons shown, one of them during revision because its estimate applied only to people aged 15–64 years, and five studies were included from the original search. Because de-duplication was performed after the title-level screening, the initial count of 389 includes duplicate records. Citation searching on 15 October 2026 identified three reports: one was an included study [55], and two were excluded after full-text assessment [66,67]. The rerun of the database searches on the same date (427 records before de-duplication) identified no additional eligible study. The supplementary PubMed search without the older-adult block (22 October 2026) identified 1051 additional records; 90 were screened on title and abstract, and three were assessed in full text, of which one was a new eligible study [51], one was an additional report of an included study [68], and one was excluded [69]. In total, six studies (seven reports) were included; three contributed to the primary meta-analysis and three to sensitivity analyses only. *n*, number of records, reports, or studies. Solid arrows show the flow of records between stages. Boxes with dashed borders show the searches carried out during peer review—the rerun of the database searches and the supplementary PubMed search—which are presented separately from the original search. Studies named in the figure: Gao et al. (2024) [52], Liu et al. (2024) [53], Tao et al. (2025) [54], Xu et al. (2019) [55], Li et al. (2026) [51], Tao et al. (2026) [68], and Kim et al. (2015) [69].

Figure 1 shows the flow of records through the review. The original database searches returned 389 records (PubMed, 225; Web of Science Core Collection, 32; ProQuest Dissertations & Theses Global, 29; APA PsycINFO, 103). Title-level screening within each database export removed 324 records that were clearly unrelated to the review question, leaving 65. Thirty-six of these were duplicates, so 29 records were screened on title and abstract, and 12 were excluded as not relevant to the review question. Full-text reports were sought for the remaining 17 records, and all 17 were retrieved and assessed for eligibility. The rerun of the database searches on 15 October 2026 retrieved 427 records before de-duplication (PubMed, 237; Web of Science Core Collection, 38; ProQuest Dissertations & Theses Global, 25 and 9 with the two variants of the strategy; EBSCOhost Complete Package, 118; Supplementary Table S2, Part B), and screening of these records against the original set identified no additional eligible study. Citation searching identified three further reports, one from the reference list of each of three included studies [52,53,54]. One of them was an included study [55]; the other two were assessed in full text and excluded (Supplementary Table S6). A multicity time-series study from Jiangsu Province, China, reported dementia mortality at the 97.5th percentile of daily mean temperature relative to the minimum-mortality temperature, derived from a continuous temperature–mortality function, and therefore provided no estimate for a defined heatwave or extreme-heat episode [66]; a study of heatwaves and mortality in 53 communities in Korea and Japan reported no dementia-specific estimate [67]. The supplementary PubMed search without the older-adult block retrieved 1051 records that the harmonized PubMed strategy had not retrieved (Supplementary Table S2, Part C). Of these, 961 did not mention mortality or death in any searchable field and were excluded, and 90 were screened on title and abstract: five were reports already assessed in this review [7,50,53,55,66], 82 were excluded, and three were assessed in full text. One study met the eligibility criteria and was included: a time-stratified case-crossover study of 64,226 dementia deaths recorded in the Jiangsu provincial mortality surveillance system in 2014–2021, which reported an OR of 1.82 (95% CI 1.61–2.06) for heatwave days (daily mean temperature at or above the 92.5th percentile for at least four days; cumulative lag 0–14 days) [51]. A second report came from the research group and the Shanghai data source of an included study and analyzed 931 deaths coded F00–F09 in 2016–2019, with heatwaves defined by a health-based excess heat factor; because these deaths are a subset of those analyzed in the included study [54], it was treated as an additional report of that study, and its estimates are listed in Supplementary Table S8 [68]. The third report estimated the change in mortality per 1 °C above a temperature threshold and did not report dementia separately, and was excluded [69]. Five reports already assessed in the review were among the records removed by the older-adult block, and one eligible study was found only without it; the block therefore reduced the sensitivity of the PubMed strategy, and the other databases were not searched again without it (Section 4.5).

Twelve of the 17 full-text reports were excluded; each is listed with its reason in Supplementary Table S6. One was a narrative review [11], and one did not concern people with dementia or exposure to ambient heat [13]. Five were excluded because they did not estimate the mortality effect of a defined heatwave among people with dementia [5,6,35,42,46]: these studies evaluated dementia or cognitive impairment as a susceptibility or effect-modifying factor in case-only or interaction-based analyses, or used exposure contrasts other than a heatwave event. Two of them address heat and mortality in people with dementia directly and are considered in the Discussion: a German health-claims cohort that reported an interaction between dementia and exposure to at least one hot day in a Cox model, without a confidence interval for the heat effect among people with dementia [46], and a primary-care cohort in England that estimated the change in mortality per 1 °C above a temperature threshold [35].

Three further reports were excluded because their results could not be expressed as a relative effect estimate with a measure of uncertainty [7,45,70]: they reported absolute excess deaths or marginal changes in the probability of death. One of these, a German health-claims study of adults aged 50 years or older, examined temperature and mortality among people with dementia directly but reported changes in percentage points per additional day in a temperature band rather than a ratio measure for a heatwave [45]. One report was excluded because it re-used the dataset and the estimates of an included study [34]. One study from Adelaide, Australia, which had been included in the initial submission, was excluded during revision because its dementia mortality estimate applied only to people aged 15–64 years and therefore did not concern the target population of older adults [50]. Together with the study identified by the supplementary PubMed search [51], six studies therefore met the eligibility criteria as applied [49,51,52,53,54,55], described in seven reports [49,51,52,53,54,55,68]; three contributed to the primary meta-analysis [49,51,55], and three to sensitivity analyses only [52,53,54] (Section 3.4).

### 3.2. Study Characteristics

The six included studies are described in Table 1; Table 2 gives, for each study, the original effect measure, the selected estimate and where it is reported, the exposure contrast, the lag period, the adjustment variables, and the role of the study in the synthesis. All six studies came from China or Australia and drew on large administrative sources—population-based mortality surveillance or linked hospital and death records. In five studies, dementia-related deaths were identified from ICD-10 cause-of-death codes in population mortality records, stated to be the underlying cause in two [52,53] and not specified further in three [49,51,54]; the sixth linked hospital-admission records with a death registry to identify deaths from Alzheimer’s disease within two months of discharge among patients who had been hospitalized for Alzheimer’s disease [55]. The dementia codes differed between studies (Table 1): one definition was confined to the registered diagnoses [55], three also included G31 [49,52,53], one used the block F00–F09 [54], and one reported dementia as the sum of Alzheimer’s disease and non-Alzheimer dementias, with the codes listed only in the supplementary material of the original article [51]. Temperature was characterized by the daily mean or by nighttime and daytime heat-excess indices, and in every study, it was assigned from outdoor ambient temperature—at the residential address in three studies [49,51,53], at county level in one [52], and from a city-wide series in two [54,55]. The heatwave definitions differed in threshold and minimum duration: location-specific percentiles combined with two to four consecutive days were most common; one study selected its definition (92.5th percentile for at least four days) from 15 candidates with the Akaike information criterion [51], and one used an intensity-based excess measure without a duration criterion [52]. The studies included between 307 and 399,036 dementia-related deaths. All six studies used a time-stratified case-crossover design and reported ORs from conditional logistic regression; none reported hazard ratios. With regard to age, two groups of studies are distinguished throughout this review: one study was restricted by design to adults aged 60 years or older [49], whereas the other five were not age-restricted; four of these were documented to be predominantly older [51,52,53,55], and one did not report the age distribution of dementia deaths [54]. Age-specific estimates for the selected definitions were reported in three studies: 1.09 (95% CI 0.67–1.77) below 65 years, 1.61 (1.13–2.28) at 65–74 years, and 1.66 (1.44–1.91) at 75 years or older in one [52]; 1.73 (1.54–1.95) below 85 years and 1.77 (1.58–1.99) at 85 years or older in another [53]; and 1.29 (1.21–1.37) at 60–74 years and 1.27 (1.23–1.30) at 75 years or older in a third [49] (Supplementary Table S8). The mapping of populations and data sources (Supplementary Table S4) identified two pairs of studies drawing on the same data source. Two studies used the China Cause of Death Reporting System (CDRS) for mainland China in overlapping years (2013–2019 [52] and 2013–2020 [49]); the study that was restricted to adults aged 60 years or older and had the longer study period and the larger number of deaths [49] was included in the primary analysis. Two studies used the Jiangsu provincial mortality surveillance system in overlapping years (2015–2020 [53] and 2014–2021 [51]); the study with the longer study period and the larger number of dementia deaths [51] was included in the primary analysis, and the other [53] was used in sensitivity analyses. The Shanghai study [54] drew on a separate municipal surveillance system but was not pooled in the primary analysis because of its outcome definition and estimand (Section 2.7); a later report from the same research group and data source used the same F00–F09 definition and was treated as an additional report of that study [68]. Because the national reporting system draws on deaths from all provinces, some deaths in the provincial and municipal datasets may also be counted in the national datasets. This partial overlap could not be quantified directly; however, even if every dementia death in the Jiangsu dataset of the primary analysis [51] were also contained in the national dataset [49], these deaths would amount to at most 16.1% of the deaths in [49] (64,226 of 399,036), and the influence of this possible dependence was examined in sensitivity analyses (Section 3.4). None of the included studies reported an estimate for non-dementia or all-cause mortality under the same exposure contrast.

### 3.3. Risk of Bias

Table 3 presents the adapted ROBINS-E ratings after re-assessment against the written criteria, and Supplementary Table S3 gives the rationale for each rating. All six studies were rated as having some concerns overall; none was rated as being at low or at high risk of bias overall. Every study raised at least some concerns for confounding, exposure measurement, and post-exposure interventions, because indoor temperature, air conditioning, medication use, and protective behavior during heat were not measured in any study and exposure was assigned from outdoor ambient temperature throughout. Selection of the reported result also raised concerns in every study, since associations were estimated for several thresholds, durations, lags, or subgroups without a prespecified primary analysis. Concerns about participant selection applied to the two single-city studies [54,55], concerns about missing data to the five studies that did not describe exclusions or missing records [49,51,52,54,55], and concerns about outcome measurement to the study in which dementia was analyzed within a broader category of mental disorders [54]. These ratings bear directly on the confidence that can be placed in the pooled estimate. The time-stratified case-crossover design used in all six studies controls for time-invariant individual characteristics, long-term trend, and season by design, so the main residual concerns are time-varying confounders such as air pollution, which was included in the main model of only two studies [51,55]. Non-differential misclassification arising from the use of outdoor temperature would be expected to bias estimates toward the null, whereas selective emphasis on larger or statistically significant results would act in the opposite direction. All three studies in the primary analysis had some concerns, and the certainty of evidence was therefore rated down by one level for risk of bias (Section 3.7).

### 3.4. Primary Analysis and Sensitivity Analyses

**Figure 2:**
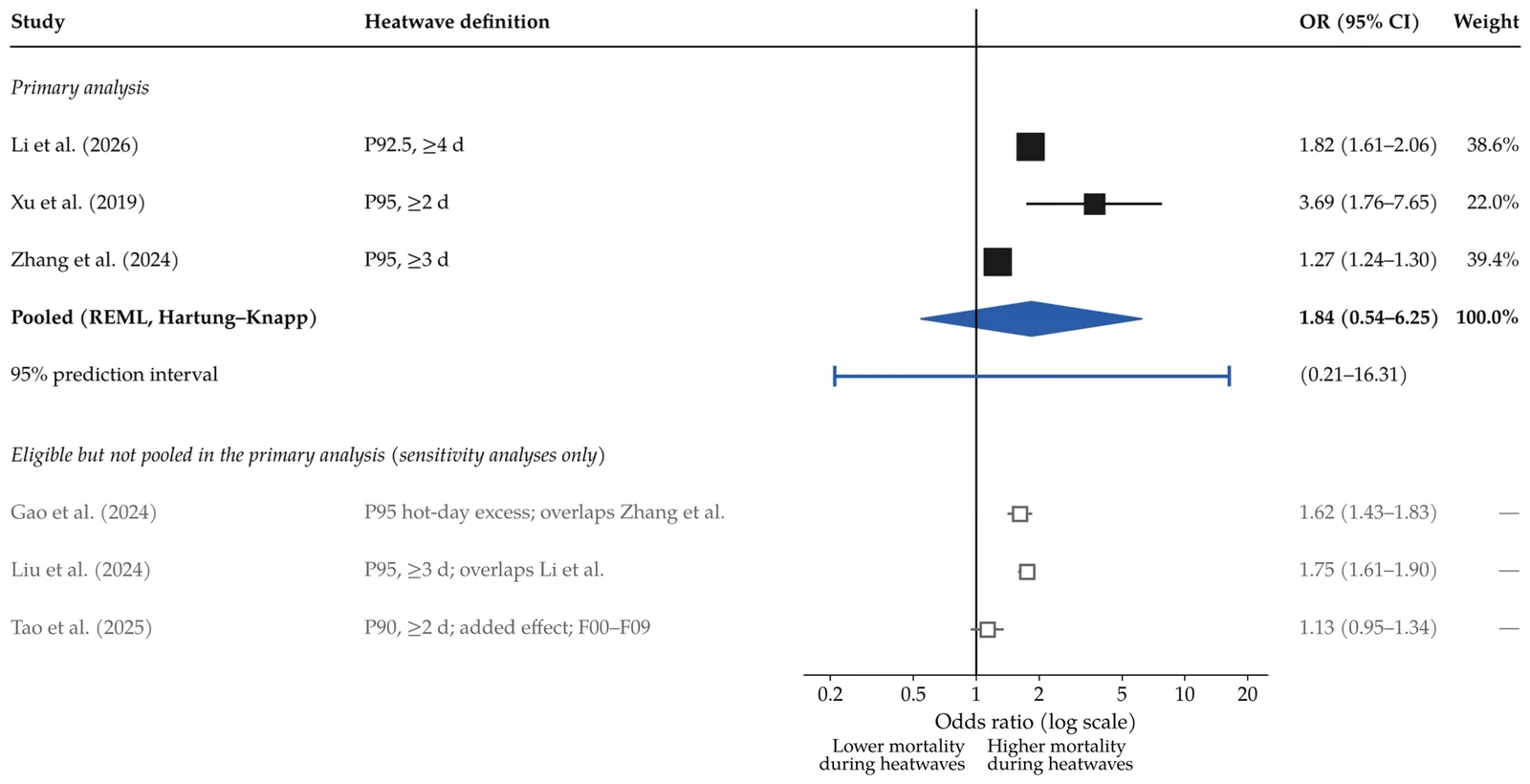
Study-specific and pooled odds ratios of dementia-related mortality during heatwave periods compared with non-heatwave periods (forest plot). Squares show study-specific odds ratios (ORs) from time-stratified case-crossover analyses, which estimate the rate ratio of death on heatwave compared with non-heatwave days; the area of each filled black square is proportional to its random-effects weight, and horizontal lines show 95% confidence intervals (CIs). The blue diamond and the bold row show the pooled estimate from the primary analysis of three studies with a dementia-specific outcome definition and an estimate of the total heatwave association (restricted maximum-likelihood estimation with the Hartung–Knapp CI), and the blue horizontal bar beneath it shows the 95% prediction interval. The open grey squares show the eligible studies that were not pooled in the primary analysis: two because their populations overlapped with those of other studies [52,53], and one because it used ICD-10 codes F00–F09 and estimated the heatwave effect conditional on temperature [54]. All study-level estimates lie to the right of the null value, but heterogeneity was high (*I*^2^ = 94.9%), and both the pooled CI and the prediction interval include the null. Studies shown: Li et al. (2026) [51], Xu et al. (2019) [55], and Zhang et al. (2024) [49] (primary analysis); Gao et al. (2024) [52], Liu et al. (2024) [53], and Tao et al. (2025) [54] (sensitivity analyses only). P90, P92.5, and P95, 90th, 92.5th, and 95th percentiles of the local temperature distribution; d, consecutive days.

Three studies were pooled in the primary analysis [49,51,55]: those with a dementia-specific outcome definition and an estimate of the total heatwave association, after one study from each of the two pairs based on the same mortality data source had been set aside [52,53]. Each of the three study-level estimates was above unity and individually statistically significant; the pooled association, however, was imprecise and did not reach statistical significance (pooled OR 1.84, 95% CI 0.54–6.25; *p* = 0.164). Figure 2 displays the study-specific and pooled estimates together with the three eligible studies that were not pooled in the primary analysis [52,53,54].

Heterogeneity among the studies in the primary analysis was high (*Q* = 39.60, *df* = 2, *p* < 0.001; *I*^2^ = 94.9%; *τ*^2^ = 0.176). The 95% prediction interval extended from 0.21 to 16.31, so the true association in a comparable new setting could plausibly lie anywhere between lower mortality and several-fold higher mortality during heatwaves. The pooled OR should therefore be read as an average across highly heterogeneous settings rather than as an effect that is expected to be observed consistently.

Table 4 summarizes the leave-one-out and sensitivity analyses. Because the primary analysis contained three studies, each leave-one-out analysis left two studies; the Hartung–Knapp CIs of these and of the other two-study analyses are based on a *t* distribution with one degree of freedom and are uninformative, and their point estimates (1.51–2.43) are reported for completeness only. *Population overlap and residual dependence.* Excluding the national dataset [49] left the provincial study [51] and the post-discharge cohort [55] (OR 2.35; 95% CI 0.03–176.78). Substituting the alternative provincial study [53] for [51] gave an OR of 1.81 (95% CI 0.54–6.12). Substituting the alternative national study [52] for [49] gave an OR of 1.76 (95% CI 1.13–2.72; *p* = 0.031), and substituting both alternatives gave 1.72 (95% CI 1.35–2.19); in these two analyses, the REML estimate of the between-study variance was close to zero (*τ*^2^ = 0.005 and 0.000), although *I*^2^ was 65.6% and 61.7%, so the narrower CIs reflect the instability of variance estimation with three studies rather than greater consistency of the evidence. Refitting the primary model with an assumed correlation of 0, 0.2, 0.4, 0.6, or 0.8 between the estimates of the national [49] and provincial [51] datasets left the pooled OR at 1.84 (95% CI 0.59–5.74 in the multivariate model, whatever the assumed correlation), because the between-study variance was much larger than the sampling variances of these two large studies; residual dependence of the extent that is possible here therefore has a negligible influence on the pooled estimate and its uncertainty. *Outcome definition and estimand.* Restricting the synthesis to the two studies of population-based dementia-coded mortality [49,51] gave an OR of 1.51 (95% CI 0.15–15.00); their individual estimates were 1.27 (95% CI 1.24–1.30) and 1.82 (95% CI 1.61–2.06). The two Alzheimer’s disease-specific estimates [51,55] gave 2.43 (95% CI 0.06–105.44). Adding the study that used ICD-10 codes F00–F09 [54], presented here as indirect evidence, gave 1.59 (95% CI 0.77–3.28) with its added effect and 1.70 (95% CI 0.92–3.14) with its intensity-related effect, and the primary analysis of the first revision gave 1.56 (95% CI 0.77–3.18). *Age.* Only one study was restricted by design to adults aged 60 years or older [49] (OR 1.27, 95% CI 1.24–1.30), so a pooled estimate limited to age-restricted populations could not be calculated; omitting the provincial study of all adult ages [51] left two studies (OR 2.03; uninformative CI). *Estimate selection.* Using the alternative estimates for [49] gave pooled ORs of 1.84 and 1.81. *Statistical method.* The CI excluded the null when all six eligible estimates were pooled irrespective of overlapping data and outcome definition (OR 1.57, 95% CI 1.15–2.15; *p* = 0.014) and when the conventional DerSimonian–Laird estimator with a Wald-type CI was used (primary set: OR 1.73, 95% CI 1.21–2.47; five studies of the initial submission with the initially selected estimates, as in the initial analysis: OR 1.50, 95% CI 1.16–1.94). With the same five initially selected estimates, the Hartung–Knapp adjustment gave CIs of 0.95–2.37 with the DerSimonian–Laird estimator and 0.94–2.43 with REML. Across the sensitivity analyses that retained at least three studies, the pooled ORs ranged from 1.50 to 1.84. The direction of the pooled estimate was therefore stable, but whether the association was statistically distinguishable from the null depended on which studies were combined, on the handling of overlapping populations, and on the random-effects method.

### 3.5. Assessment of Small-Study Effects

Small-study effects could not be evaluated in a meaningful way. With three studies in the primary analysis and six overall, and with high heterogeneity, asymmetry in a funnel plot cannot be told apart from genuine between-study differences, and regression-based tests such as Egger’s are unreliable with fewer than 10 studies [64]. Supplementary Figure S1 shows the funnel plot of the six study-level estimates for completeness; the smallest study [55] reported the largest estimate, a pattern that is compatible with small-study effects or selective reporting but equally with real differences between settings. The funnel plot in the initial submission had been exported from the meta-analysis software with the trim-and-fill option enabled and therefore displayed imputed studies and an adjusted estimate that were not described in the text. That procedure had not been planned and is not reliable with few, highly heterogeneous studies. When it was reproduced on the initially selected estimates of the five studies of the initial submission that remain eligible, two studies were imputed, and the adjusted pooled estimates were 1.15 (95% CI 1.13–1.17) under a fixed-effect model and 1.21 (95% CI 0.95–1.55) under a random-effects model (Supplementary File S1); these values are reported for transparency only and were not used for inference. Whether publication bias is present could not be established from the available evidence.

### 3.6. Descriptive Stratification by Study Characteristics

Because the categories of country, heatwave threshold, and duration each contained few studies, formal subgroup analyses were not performed; Table 5 presents the study-level estimates descriptively. The Australian estimate (3.69) was larger than the five Chinese estimates (1.13–1.82). This contrast cannot be attributed to geography, because country was confounded with other study characteristics: the Australian estimate came from the smallest study, which also differed from the Chinese studies in outcome definition (Table 5). Among the percentile-based definitions, the single estimate at the 90th percentile (1.13) was lower than the estimates at the 92.5th and 95th percentiles (1.27–3.69), but this comparison rests on one study that also differed in its outcome coding and in estimating the heatwave effect conditional on temperature [54]. Heatwave duration, a registered secondary objective, could not be examined as a subgroup, because the duration categories were confounded with the other characteristics; the estimates reported within studies for different thresholds and durations are listed in Supplementary Table S8. No conclusion about differences between countries, thresholds, or durations can therefore be drawn from the available studies.

### 3.7. Certainty of Evidence

The certainty of evidence for the association between heatwave exposure and dementia-related mortality was rated as very low (Table 6). Starting from low certainty for observational evidence, the rating was lowered for risk of bias (some concerns in all three studies in the primary analysis, chiefly for confounding, exposure measurement, and selection of the reported result), inconsistency (*I*^2^ = 94.9% and a 95% prediction interval of 0.21–16.31), indirectness (differences among studies in outcome definition, dementia coding, and age range; only one study restricted to adults aged 60 years or older; and evidence from only two countries), and imprecision (a CI that includes both no association and a sixfold increase). Publication bias could not be assessed. No criterion for rating up was met: the pooled estimate was below 2, and an exposure–response gradient could not be assessed reliably. The rating does not depend on the risk-of-bias judgment: without rating down for risk of bias, inconsistency and imprecision alone would still result in very low certainty.

## 4. Discussion

This systematic review and meta-analysis synthesized the available evidence on the association between heatwave exposure and dementia-related mortality. Six eligible studies conducted in China and Australia were identified, and every study-level estimate indicated higher mortality during heatwaves. However, when the synthesis was restricted to the three studies with a dementia-specific outcome definition and an estimate of the total heatwave association, with one study from each pair based on the same mortality data set aside, and methods suited to a small number of heterogeneous studies were used, the association was imprecise and not statistically significant (pooled OR 1.84, 95% CI 0.54–6.25), heterogeneity was high (*I*^2^ = 94.9%), and the 95% prediction interval (0.21–16.31) was wide. Because all estimates were conditional ORs from case-crossover analyses, which estimate rate ratios, the pooled OR can be read as the average relative change in the dementia-related mortality rate during heatwaves. A statistically significant pooled estimate was obtained only when all eligible estimates were pooled even though two pairs of them drew on the same mortality data, when the alternative national study was substituted in analyses in which the between-study variance was estimated to be close to zero from three studies, or when a random-effects method that can produce overly narrow CIs with few studies was used (Table 4); such results should not be regarded as an established effect size. Evidence from the general older population has similarly shown increased heat-related mortality [71], and future heat-related mortality is projected to rise substantially as populations age [72]. None of the included studies compared the relative increase in dementia deaths with that in deaths from other causes; the present synthesis therefore cannot show that people with dementia are more susceptible to heat than other older adults, only that dementia-coded deaths, like deaths in the older population generally, tend to be more frequent during heatwaves. Taken together, the findings are inconclusive regarding the magnitude and consistency of the association: the study-level estimates share a direction, but the pooled estimate is too imprecise to establish an effect, its presence in any given setting remains uncertain, and it is an average across highly heterogeneous settings rather than a universally transferable effect size.

### 4.1. Comparison with Existing Evidence

The direction of the study-level estimates, although not the precision of the pooled estimate, is consistent with the broader literature on heat and dementia. Population-based studies in China have repeatedly reported higher dementia-related mortality during heatwaves [49,51,52,53], as did one study of a broader diagnostic category [54], and a recent meta-analysis of studies in adults aged 60 years or older reported a pooled RR of 1.27 (95% CI 0.96–1.68) for mortality related to mental disorders on heatwave days, based on four studies [47]—an estimate that, like ours, pointed toward harm without excluding the null. Whether heat affects people with dementia more than other older adults is a separate question that the included studies cannot answer, because none of them reported a comparison group. The comparative evidence comes from studies that were not eligible for pooling. In German health-claims data for people aged 65 years or older, exposure to at least one hot day was not associated with mortality among people without dementia (hazard ratio 0.98) but interacted with dementia (interaction hazard ratio 1.11, *p* = 0.011) [46]; a later German study reported that heat raised the probability of death only among people with dementia [45]; and a case-only analysis of deaths among Medicare beneficiaries in 135 cities in the United States estimated that the risk of dying on an extremely hot day was a further 8% (95% CI 4–12%) higher among people previously admitted for Alzheimer’s disease and 6% (95% CI 3–9%) higher among those previously admitted for dementia [5]. In a primary-care cohort of patients with psychosis, dementia, or substance misuse in England, mortality rose by 4.9% per 1 °C above the regional temperature threshold overall, with the greatest risk among younger patients and those with substance misuse rather than among those with dementia [35]. These findings suggest, but do not establish, greater susceptibility among people with dementia. Our results also align conceptually with the parallel body of work showing that heat elevates dementia-related hospitalizations and emergency care [16,17,18,19,20,21,22] and is associated with cognitive decline [23,24,25,26,27]. Recent modeling further projects that dementia deaths attributable to extreme temperature events will rise as the climate warms and populations age [34]. The present review complements these studies by bringing together heatwave-related mortality estimates for dementia specifically and by making explicit how little independent evidence currently underlies them.

### 4.2. Potential Mechanisms

The mechanisms outlined below are biologically plausible explanations for an association between heat and mortality in dementia; they were not tested in the included studies. Physiologically, dementia-related neurodegeneration may disrupt hypothalamic thermoregulation, autonomic cardiovascular control, and thirst perception, reducing the capacity to dissipate heat and maintain fluid balance during thermal stress [11,13,14]. Behaviorally, changes in memory, judgment, and communication can make it harder to recognize heat, hydrate, remove clothing, operate cooling devices, or seek help, so protective actions may be delayed or may not occur without support [12,31]. Pharmacological factors may add to this vulnerability, because antipsychotic, anticholinergic, and sedative medications prescribed to some people with dementia can impair sweating and thermoregulatory responses [15,41]. Finally, contextual and social determinants, including reliance on caregivers, residential care, social isolation, and socioeconomic disadvantage, shape both exposure and the ability to respond and may amplify biological susceptibility in the most disadvantaged groups [42,43,44]. These pathways are likely to be mutually reinforcing rather than mutually exclusive, and their relevance varies from person to person with the type and stage of dementia and with the support available.

### 4.3. Heterogeneity and Exploratory Findings

The high heterogeneity may reflect differences across the included studies in geographic setting, climate, heatwave definition, dementia ascertainment, outcome definition, study design, and population age structure, and it could not be attributed to any single study. The descriptive stratification offers only limited insight. The Australian estimate was larger than the Chinese estimates, and the estimate at the 90th-percentile threshold was lower than that at the 95th percentile; however, the categories contained as few as one study, and country, threshold, outcome definition, age range, and study size varied together. In particular, the larger Australian estimate came from a cohort of patients recently discharged after hospitalization for Alzheimer’s disease, and so cannot be attributed to geography; we therefore do not offer an explanation for the difference between the Australian and Chinese estimates. These observations should be regarded as hypothesis-generating rather than confirmatory, and consistent, prospectively harmonized heatwave definitions are needed before firm conclusions can be drawn [30,39,40].

### 4.4. Public Health and Policy Implications

The certainty of the evidence is very low, the prediction interval includes the null, the evidence comes from two countries, and no study compared dementia with other deaths. The findings therefore do not, on their own, justify dementia-specific policy recommendations, and we limit ourselves to two observations. First, the direction of the study-level estimates is consistent with the well-documented heat-related mortality among older adults in general [4,71], so existing guidance to include older people with care needs—among them people with dementia—in heat–health action plans and early-warning systems is not contradicted by the available evidence [4,15]. These implications also need to be read in light of the age composition of the evidence: only one study was restricted by design to adults aged 60 years or older [49], and its estimate (OR 1.27) was the smallest of the dementia-specific estimates, whereas the other studies included all adults or all ages and were predominantly, but not exclusively, older, or did not report age [54]. Implications for older adults with dementia therefore rest largely on evidence from predominantly older rather than exclusively older populations. Second, because people with dementia form a large and growing population [9], even a modest relative increase would matter in absolute terms, which makes better primary evidence a priority. Specific measures that have been proposed, such as cooling standards in care settings, medication review during hot weather, hydration support, and caregiver education [11,20,31,41], rest on mechanistic reasoning and on studies of other outcomes rather than on the mortality evidence synthesized here, and their effect on deaths among people with dementia has not been evaluated.

If such measures are pursued, they are unlikely to work as isolated sectoral responses. In aging societies, the capacity to protect older adults with complex care needs during heat events depends on the healthcare workforce, long-term care, housing, transport, social support, and the availability of caregivers, and recent work on demographic transformation argues that the challenges of population aging call for integrated approaches that involve health and social services, urban planners, policymakers, older adults, and caregivers, with explicit attention to social determinants [73]. Heat-related mortality in dementia illustrates this argument: any reduction is likely to depend on coordinated action across healthcare, social care, housing, and community support—for example, linking heat alerts to home-care and long-term care services and involving people living with dementia and their caregivers in local planning—rather than on a single clinical intervention. This systems perspective is offered as a framework for future policy and research, not as a conclusion drawn from our data.

### 4.5. Strengths and Limitations

The strengths of this review include public registration, reporting according to PRISMA 2020, a rerun of the searches with harmonized strategies supplemented by citation searching and by a PubMed search without the older-adult block, use of an adapted ROBINS-E assessment and GRADE, explicit mapping of overlapping populations with a quantitative assessment of residual dependence, a transparent rule for selecting among multiple estimates, random-effects methods suited to a small number of heterogeneous studies, and open provision of the study-level data and analysis code. Several limitations should nonetheless be acknowledged; several of them were raised during peer review and are stated explicitly here because the available data allowed them to be mitigated but not removed. First, only six studies met the eligibility criteria, all conducted in China and Australia, and only three of them met the conditions for the primary analysis; this limits precision and geographic generalizability and precludes meta-regression and any assessment of small-study effects. Second, between-study heterogeneity was high, and both the pooled CI and the prediction interval included the null value; the pooled estimate is therefore a summary of the average association across the included studies rather than a universally applicable effect. Third, the evidence is indirect with respect to the registered review question. Only one study was restricted to adults aged 60 years or older; the others were not restricted by age and were predominantly older or did not report the age distribution of dementia deaths. Because the age criterion was defined by the age range to which an estimate referred, estimates for all ages or all adults were eligible; they include an unknown share of deaths before the age of 60 years that could not be separated, and their eligibility rests on the expectation, rather than the demonstration, that older adults accounted for most of the deaths. Most studies analyzed dementia-coded deaths in mortality records, which describes the occurrence of dementia-coded deaths during heatwaves rather than the risk of death among living people with a dementia diagnosis, and cause-of-death coding of dementia is subject to under-reporting and misclassification [49,52]. The primary analysis combined these population-based estimates with one estimate from a cohort of patients discharged after hospitalization for Alzheimer’s disease; the two outcome types do not identify the same epidemiological quantity, and the restricted synthesis of the population-based studies alone was too small to be informative. Expressing all estimates as ORs from case-crossover analyses does not make them epidemiologically equivalent; combining them in one primary analysis is a judgment that other reviewers might not share, and the primary pooled estimate should be read as a summary of related but non-identical quantities. The dementia definitions of most studies were broader than the registered diagnostic categories, and one study used a diagnostic block that includes conditions other than dementia and estimated the heatwave effect only conditional on temperature; it was therefore presented only as indirect evidence, and its inclusion in sensitivity analyses lowered the pooled estimate (OR 1.59 or 1.70, depending on the estimate used). No study provided a comparison with non-dementia deaths, so relative susceptibility could not be assessed. Fourth, the review was registered after the searches had been run and screening had begun, and several features were decided or changed post hoc: the age criterion was set with knowledge of the candidate studies and reformulated during peer review, study authors were not contacted for unreported data, heatwave duration was not analyzed as planned, one study was excluded during peer review because its estimate applied only to people younger than 65 years, and the primary analysis set, the estimate-selection hierarchy, the statistical method, and the risk-of-bias ratings were re-specified during peer review after the initial results were known; the primary analysis set was re-specified again in the second round of review (Supplementary Table S7). Fifth, the independence of the estimates in the primary analysis could not be fully established. Two pairs of studies drew on the same mortality data source, and only one study from each pair was retained, but partial overlap between the national dataset and the provincial dataset in the primary analysis is likely; it could not be quantified directly, but it was bounded at 16.1% of the national deaths and had a negligible influence on the pooled estimate in a model allowing for correlated estimates, which nevertheless cannot establish that the estimates are fully independent. Both datasets were retained because any overlap was bounded in this way and because excluding either would have reduced the primary analysis to two studies; however, the pooled estimate depended on which of them was omitted (OR 2.35 without the national dataset and 2.03 without the provincial dataset, both with uninformative CIs), so the primary estimate is not robust to this choice. Sixth, the search had limitations. In the original search, only the PubMed strategy was saved verbatim, the strategies were not harmonized across databases, and de-duplication followed an initial title-level screening, so the number of unique records retrieved is not available. The rerun with harmonized strategies, carried out during peer review, improved the reproducibility of the strategies, but the number of records removed or excluded at each step of the rerun was not documented, so the conclusion that the rerun identified no further eligible study cannot be verified step by step. The strategies included a concept block for older adults; a supplementary PubMed search without this block identified one further eligible study and one additional report of an included study, which shows that the block reduced sensitivity, and because the other databases were not searched without it, further studies indexed only in those databases may have been missed. Embase and Scopus were not searched; gray literature was covered only through a dissertation database; and only English-language publications were eligible. Seventh, risk of bias was rated with an adapted ROBINS-E approach—review-specific criteria for each domain rather than the full set of signaling questions and domain algorithms—so the judgments are not equivalent to those of a complete ROBINS-E assessment; the overall judgment for each study was taken as its most severe domain-level judgment, and the ratings were revised during peer review. Eighth, the included studies used heterogeneous heatwave definitions, including one selected by the original authors from 15 candidate definitions [51], and assigned exposure from outdoor ambient temperature, and residual misclassification of exposure and dementia status cannot be excluded. Finally, all included studies had at least some concerns in the risk-of-bias assessment. These limitations underlie the very low certainty rating and reinforce the need for cautious interpretation and for additional, methodologically harmonized primary studies.

## 5. Conclusions

This systematic review and meta-analysis found that the evidence on heatwaves and dementia-related mortality is inconclusive regarding the magnitude and consistency of the association. All study-level estimates pointed toward higher mortality during heatwaves, but in the primary analysis of three studies with a dementia-specific outcome definition and an estimate of the total heatwave association, the pooled OR was 1.84 (95% CI 0.54–6.25), with high heterogeneity and a prediction interval spanning the null, and the certainty of evidence was very low. The pooled estimate represents an average across highly heterogeneous settings, and a harmful association was not demonstrated consistently across comparable populations; apparent differences between countries and heatwave thresholds rest on one or two studies each and cannot be interpreted. Because no study compared dementia with other deaths, the evidence does not show whether people with dementia are more susceptible to heat than other older adults, and because only one study was restricted to adults aged 60 years or older, whereas the others were predominantly older or did not report age, the evidence for older adults with dementia is largely indirect.

These findings do not support dementia-specific policy recommendations beyond existing guidance for older adults with care needs. The consistent direction of the study-level estimates and the plausibility of the mechanisms are compatible with including people with dementia in heat–health action plans as a precaution [4,15], ideally within integrated strategies for aging populations [73], but the effect of specific measures on mortality has not been evaluated. Because the current evidence base is small, geographically narrow, partly overlapping, and highly heterogeneous, future research should prioritize studies from underrepresented regions and climates, report age-specific estimates for older adults, follow defined cohorts of people living with dementia in addition to analyzing cause-of-death records, include comparison groups without dementia, adopt harmonized heatwave definitions with prespecified primary estimates, and, once enough independent studies are available, use meta-regression to investigate sources of heterogeneity.

## Figures and Tables

**Table 1:** Characteristics of the included studies.

Study	Region	Study Population	Dementia Ascertainment	Mortality Data Source	Temperature Indicator	Heatwave Definition	*n*
Gao et al. (2024) [52]	Mainland China	Decedents of all ages whose underlying cause of death was dementia, May–September 2013–2019; mean age 82.5 years; 81.6% aged ≥75 years	ICD-10 codes F00–F03, G30, and G31 recorded as the underlying cause of death	China Cause of Death Reporting System (CDRS)	Hourly air temperature used to derive hot-night excess (HNE) and hot-day excess (HDE); daily mean, minimum, and maximum temperatures were also assessed	HNE and HDE defined using local 90th, 92.5th, 95th, and 97.5th percentile thresholds for nighttime minimum and daytime maximum temperature, respectively	132,573
Li et al. (2026) [51]	Jiangsu Province, China	Adults whose cause of death was dementia (Alzheimer’s disease or non-Alzheimer dementias), 2014–2021; 72.5% of dementia deaths at >80 years	Cause of death coded according to ICD-10 (codes listed in the supplementary material of the original article)	Mortality Surveillance System of the Jiangsu Provincial Center for Disease Control and Prevention	Daily mean temperature from ERA5-Land reanalysis (0.1° × 0.1°) assigned according to residential address	Daily mean temperature (May–September) ≥90th, 92.5th, 95th, 97.5th, or 99th percentile for ≥2, ≥3, or ≥4 consecutive days; ≥92.5th percentile for ≥4 d selected by the original authors (Akaike information criterion)	64,226
Liu et al. (2024) [53]	Jiangsu Province, China	Adults aged ≥18 years whose underlying cause of death was dementia, 2015–2020; mean age 84.2 years (SD 9.2)	Dementia identified from death certificates using ICD-10 codes F01, F03, G30, and G31	Jiangsu Provincial Mortality Surveillance System	Gridded daily 24-h mean temperature assigned according to residential address	Daily mean temperature exceeding the grid-specific 90th, 92.5th, 95th, or 97.5th percentile for ≥2, ≥3, or ≥4 consecutive days	57,791
Tao et al. (2025) [54]	Shanghai, China	Decedents of all ages who died from dementia during the warm season (May–September), 2008–2021; age distribution of dementia deaths not reported	Deaths coded F00–F09 (organic, including symptomatic, mental disorders), designated as dementia by the study authors	Shanghai Municipal Center for Disease Control and Prevention mortality surveillance data	Daily mean temperature calculated from hourly temperature data	Daily mean temperature exceeding the 90th, 95th, or 99th percentile for ≥2, ≥3, or ≥4 consecutive days; main analyses used 90p_2d, 95p_3d, and 99p_4d	3322
Xu et al. (2019) [55]	Brisbane, Queensland, Australia	Patients hospitalized for Alzheimer’s disease (2005–2013) who died from Alzheimer’s disease within two months after discharge; 98.4% of decedents aged ≥65 years	Alzheimer’s disease identified from hospital admission records linked to death-registry records, with Alzheimer’s disease recorded as the underlying cause of death (ICD codes not reported)	Registrar General Death Database linked to the Queensland Hospital Admitted Patient Data Collection	Daily mean temperature calculated as the average of daily maximum and minimum temperatures	Daily mean temperature at or above the 90th, 95th, or 97th percentile for ≥2 consecutive days	307
Zhang et al. (2024) [49]	Nationwide, China	Adults aged ≥60 years whose underlying cause of death was Alzheimer’s disease or another dementia, 2013–2020	ICD-10 codes F01, F03, G30, and G31 recorded as the cause of death	China Cause of Death Reporting System (CDRS)	Daily mean temperature interpolated to each decedent’s residential location	Location-specific daily mean temperature at or above the 90th, 95th, or 97.5th percentile for ≥2, ≥3, or ≥4 consecutive days	399,036

Abbreviations: CDRS, China Cause of Death Reporting System; HDE, hot-day excess; HNE, hot-night excess; ICD-10, International Classification of Diseases, 10th Revision; *n*, number of dementia-related deaths in the study. Studies are listed in alphabetical order of the first author’s surname.

**Table 2:** Original effect measures, selected estimates, and role of each study in the synthesis.

Study	Design and Model	Original Effect Measure	Selected Estimate (95% CI) and Its Source	Exposure Contrast for the Selected Estimate	Lag	Adjustment Variables	Role in Synthesis
Gao et al. (2024) [52]	Time-stratified case-crossover; conditional logistic regression with DLNM	OR	1.62 (1.43–1.83); Table 1 in [52]	Extreme hot-day excess (95th-percentile threshold of daily maximum temperature) vs. reference excess (1st percentile, approximating the lowest risk); no duration criterion	Cumulative, 0–13 d	Daily mean temperature, relative humidity, holidays; time-invariant individual factors by design	Sensitivity analyses only (population overlaps with [49])
Li et al. (2026) [51]	Time-stratified case-crossover; conditional logistic regression with DLNM	OR	1.82 (1.61–2.06); Section 3.2 in [51]	Heatwave days (daily mean ≥92.5th percentile for ≥4 d) vs. non-heatwave days	Cumulative, 0–14 d	Air quality index, relative humidity; time-invariant individual factors, season, and day of week by design	Primary analysis
Liu et al. (2024) [53]	Time-stratified case-crossover; conditional logistic regression with DLNM	OR	1.75 (1.61–1.90); Table 2 in [53]	Heatwave days (daily mean ≥95th percentile for ≥3 d) vs. non-heatwave days	Cumulative, 0–10 d	Relative humidity; time-invariant individual factors, season, and day of week by design	Sensitivity analyses only (population overlaps with [51])
Tao et al. (2025) [54]	Time-stratified case-crossover; conditional logistic regression with time-varying DLNM	OR	1.13 (0.95–1.34); Results text in [54]	Added effect of heatwave days (daily mean ≥90th percentile for ≥2 d) vs. non-heatwave days, conditional on temperature	Same day	Daily mean temperature (intensity term), relative humidity; time-invariant individual factors by design	Indirect evidence; sensitivity analyses only (ICD-10 F00–F09; effect conditional on temperature)
Xu et al. (2019) [55]	Time-stratified case-crossover within a retrospective cohort; conditional logistic regression	OR (reported as percentage change)	3.69 (1.76–7.65); Abstract in [55], given as a 269% (76–665%) increase	Heatwave days (daily mean ≥95th percentile for ≥2 d) vs. non-heatwave days	Same day, as shown in Figure 2 in [55]; lags of 0–7 d examined	PM_10_, NO_2_, relative humidity; time-invariant individual factors by design	Primary analysis
Zhang et al. (2024) [49]	Time-stratified case-crossover; conditional logistic regression with DLNM	Cumulative OR	1.27 (1.24–1.30); Figure 2b of the original article [49]	Heatwave days (daily mean ≥95th percentile for ≥3 d) vs. non-heatwave days	Cumulative, 0–7 d	Relative humidity, atmospheric pressure, sex, age, city, holidays	Primary analysis

Abbreviations: CI, confidence interval; d, days; DLNM, distributed lag non-linear model; NO_2_, nitrogen dioxide; OR, odds ratio; PM_10_, particulate matter with an aerodynamic diameter ≤10 μm. Estimates were selected using the hierarchy described in Section 2.5; the location of each estimate in the original publication is given after the estimate (see also Supplementary Table S5). For [49], the initially selected estimate (90th percentile for ≥2 d: 1.14, 95% CI 1.12–1.16) and the estimate for the 95th percentile for ≥2 d (1.25, 95% CI 1.22–1.28) were used in sensitivity analyses. For [51], only the definition selected by the original authors was reported numerically; its estimate for Alzheimer’s disease deaths (1.97, 95% CI 1.59–2.44) was used in a sensitivity analysis.

**Table 3:** Adapted ROBINS-E domain and overall ratings of the included studies after re-assessment.

Study	D1	D2	D3	D4	D5	D6	D7	Overall Judgment
Gao et al. (2024) [52]	Some concerns	Some concerns	Low risk	Some concerns	Some concerns	Low risk	Some concerns	Some concerns
Li et al. (2026) [51]	Some concerns	Some concerns	Low risk	Some concerns	Some concerns	Low risk	Some concerns	Some concerns
Liu et al. (2024) [53]	Some concerns	Some concerns	Low risk	Some concerns	Low risk	Low risk	Some concerns	Some concerns
Tao et al. (2025) [54]	Some concerns	Some concerns	Some concerns	Some concerns	Some concerns	Some concerns	Some concerns	Some concerns
Xu et al. (2019) [55]	Some concerns	Some concerns	Some concerns	Some concerns	Some concerns	Low risk	Some concerns	Some concerns
Zhang et al. (2024) [49]	Some concerns	Some concerns	Low risk	Some concerns	Some concerns	Low risk	Some concerns	Some concerns

Abbreviations: D1, confounding; D2, measurement of the exposure; D3, selection of participants; D4, post-exposure interventions; D5, missing data; D6, measurement of the outcome; D7, selection of the reported result. The overall judgment corresponds to the most severe domain-level judgment (Section 2.6). The criteria applied and the rationale for each rating are given in Supplementary Table S3.

**Table 4:** Primary analysis, leave-one-out analysis, and sensitivity analyses.

Analysis	Studies Included	*k*	Pooled OR (95% CI)	*p*-Value	*I*^2^ (%)	*τ* ^2^	95% PI
Primary analysis							
Three studies with a dementia-specific outcome definition and an estimate of the total heatwave association	[49,51,55]	3	1.84 (0.54–6.25)	0.164	94.9	0.176	0.21–16.31
Leave-one-out analysis (primary set) ^a^							
Omitting Li et al. (2026) [51] ^b^	[49,55]	2	2.03 (0.00–1703.77)	0.410	87.7	0.500	—
Omitting Xu et al. (2019) [55] ^c^	[49,51]	2	1.51 (0.15–15.00)	0.263	96.8	0.063	—
Omitting Zhang et al. (2024) [49] ^d^	[51,55]	2	2.35 (0.03–176.78)	0.241	71.1	0.178	—
Sensitivity analyses: population overlap and residual dependence							
Liu et al. [53] substituted for Li et al. [51]	[49,53,55]	3	1.81 (0.54–6.12)	0.171	96.7	0.171	0.21–15.67
Gao et al. [52] substituted for Zhang et al. [49]	[51,52,55]	3	1.76 (1.13–2.72)	0.031	65.6	0.005	1.03–3.01
Both alternative studies substituted [52,53]	[52,53,55]	3	1.72 (1.35–2.19)	0.011	61.7	0.000	1.35–2.19
Assumed correlation of 0 to 0.8 between [49] and [51] ^e^	[49,51,55]	3	1.84 (0.59–5.74)	—	—	0.176	—
Sensitivity analyses: outcome definition and estimand							
Population-based dementia-coded mortality only ^a^	[49,51]	2	1.51 (0.15–15.00)	0.263	96.8	0.063	—
Alzheimer’s disease-specific estimates (Li et al. [51]: 1.97, 95% CI 1.59–2.44) ^a^	[51,55]	2	2.43 (0.06–105.44)	0.205	61.3	0.121	—
Adding Tao et al. [54], added effect (1.13, 95% CI 0.95–1.34)	[49,51,54,55]	4	1.59 (0.77–3.28)	0.135	92.8	0.138	0.40–6.37
Adding Tao et al. [54], intensity-related effect (1.51, 95% CI 0.94–2.43)	[49,51,54,55]	4	1.70 (0.92–3.14)	0.071	92.5	0.086	0.56–5.19
Primary analysis of the first revision	[49,53,54,55]	4	1.56 (0.77–3.18)	0.138	95.3	0.127	0.41–5.95
Sensitivity analyses: estimate selection							
Zhang et al. [49]: 95th percentile, ≥2 d (1.25, 95% CI 1.22–1.28)	[49,51,55]	3	1.84 (0.53–6.37)	0.169	95.3	0.185	0.20–17.09
Zhang et al. [49]: 90th percentile, ≥2 d (1.14, 95% CI 1.12–1.16)	[49,51,55]	3	1.81 (0.46–7.15)	0.206	96.9	0.243	0.14–22.67
Sensitivity analyses: all eligible studies and statistical method							
All six eligible studies, estimates selected by the hierarchy	[49,51,52,53,54,55]	6	1.57 (1.15–2.15)	0.014	95.1	0.052	0.81–3.06
Five studies of the initial submission, initially selected estimates, REML with Hartung–Knapp CI	[49,52,53,54,55]	5	1.51 (0.94–2.43)	0.071	97.0	0.089	0.58–3.93
Five studies of the initial submission, initially selected estimates, DerSimonian–Laird with Hartung–Knapp CI	[49,52,53,54,55]	5	1.50 (0.95–2.37)	0.070	97.0	0.073	0.62–3.61
Five studies of the initial submission, initially selected estimates, DerSimonian–Laird with Wald-type CI (method of the initial analysis) ^f^	[49,52,53,54,55]	5	1.50 (1.16–1.94)	0.002	97.0	0.073	0.58–3.90
Primary set, DerSimonian–Laird with Wald-type CI ^f^	[49,51,55]	3	1.73 (1.21–2.47)	0.002	94.9	0.075	0.03–112.96

Abbreviations: CI, confidence interval; d, consecutive days; *k*, number of studies included in the analysis; OR, odds ratio; PI, prediction interval; REML, restricted maximum likelihood. Unless otherwise stated, pooled estimates were calculated using a random-effects model with REML estimation of *τ*^2^ and the Hartung–Knapp CI, and the PI was based on the *t* distribution with *k* − 1 degrees of freedom; PIs from three studies are very wide and are shown for completeness. ^a^ With two studies, the Hartung–Knapp CI is based on the *t* distribution with one degree of freedom and is uninformative, and no PI is shown. ^b^ Also corresponds to omitting the provincial study of all adult ages. ^c^ Also corresponds to the restricted synthesis of population-based dementia-coded mortality (exclusion of the post-discharge cohort). ^d^ Also corresponds to the exclusion of the national dataset. ^e^ Multivariate random-effects model (REML) with a *t*-based CI; the result was identical for every assumed correlation from 0 to 0.8. ^f^ Re-run with the CI limits as reported by the original authors; the PI was calculated as in the initial analysis, using the *t* distribution with *k* − 2 degrees of freedom.

**Table 5:** Descriptive stratification of study-level estimates by study characteristics (all six eligible studies).

Characteristic	Category	*k*	Studies	Study-Level OR (Range)
Country	Australia	1	[55]	3.69
	China	5	[49,51,52,53,54]	1.13–1.82
Heatwave threshold	90th percentile	1	[54]	1.13
	92.5th percentile	1	[51]	1.82
	95th percentile	4	[49,52,53,55]	1.27–3.69
Minimum duration	None (intensity-based excess)	1	[52]	1.62
	≥2 consecutive days	2	[54,55]	1.13–3.69
	≥3 consecutive days	2	[49,53]	1.27–1.75
	≥4 consecutive days	1	[51]	1.82
Outcome definition	Dementia-coded deaths in population mortality records	5	[49,51,52,53,54]	1.13–1.82
	Deaths from Alzheimer’s disease after discharge in a cohort hospitalized for Alzheimer’s disease	1	[55]	3.69
Age directness	Restricted to ≥60 years	1	[49]	1.27
	Not restricted; predominantly older	4	[51,52,53,55]	1.62–3.69
	Not restricted; age distribution not reported	1	[54]	1.13
Dementia definition	Confined to the registered diagnoses (Alzheimer’s disease)	1	[55]	3.69
	Also including G31	3	[49,52,53]	1.27–1.75
	Alzheimer’s disease and non-Alzheimer dementias (codes not given in the main report)	1	[51]	1.82
	Block F00–F09	1	[54]	1.13

Abbreviations: *k*, number of studies; OR, odds ratio. The table is descriptive: no pooled subgroup estimates or tests of subgroup differences are reported because categories contained few studies and study characteristics were confounded with one another. Study-level estimates are those listed in Table 2.

**Table 6:** GRADE summary of the certainty of evidence.

Outcome	Studies (Deaths)	Pooled OR (95% CI)	Risk of Bias	Inconsistency	Indirectness	Imprecision	Publication Bias	Certainty
Dementia-related mortality during heatwave vs. non-heatwave periods	3 studies in the primary analysis (463,569 deaths); 6 eligible studies	1.84 (0.54–6.25)	Serious ^a^	Very serious ^b^	Serious ^c^	Serious ^d^	Not assessable ^e^	Very low

Abbreviations: CI, confidence interval; GRADE, Grading of Recommendations Assessment, Development and Evaluation; OR, odds ratio. Evidence from observational studies starts at low certainty. ^a^ Some concerns in the adapted ROBINS-E assessment, chiefly for confounding, exposure measurement, and selection of the reported result, in all three studies; the certainty would remain very low without rating down for risk of bias. ^b^ *I*^2^ = 94.9%; 95% prediction interval 0.21–16.31. ^c^ Heterogeneous outcome definitions, dementia coding, and age ranges; one study restricted to adults aged ≥60 years; evidence from two countries only. ^d^ The CI includes both no association and a sixfold increase. ^e^ Fewer than 10 studies; not rated down.

## Data Availability

All study-level data used in the analyses and the analysis code are openly provided in the Supplementary Materials (Files S1 and S2); the data are also reported in Table 1 and Table 2. The data-extraction form is available from the corresponding author upon reasonable request.

## Supplementary Materials

The following supporting information can be downloaded at: https://www.mdpi.com/article/10.3390/jcm15218655/s1, Table S1: PRISMA 2020 checklist; Table S2: Search strategies (original search, rerun, citation searching, and supplementary PubMed search); Table S3: Adapted ROBINS-E criteria, ratings, and rationale; Table S4: Mapping of populations and data sources and assessment of overlap; Table S5: Selected estimates, their sources, and application of the estimate-selection hierarchy; Table S6: Full-text reports excluded, with reasons; Table S7: Departures from the registered record; Table S8: Numerically reported estimates for alternative heatwave definitions and age groups; Figure S1: Funnel plot of the six study-level estimates; File S1: R code for the meta-analyses; File S2: Study-level data.
