# Supplementary material for: Extreme Heat Exposure and Dementia-Related Mortality: A Systematic Review and Meta-Analysis
Source: J Clin Med. 2026 Nov 6;15(21):8655. doi: 10.3390/jcm15218655 (PMC13763779; doi:10.3390/jcm15218655)
Supplement: Supplementary file 1 [file jcm-15-08655-s001.zip › Supplementary_Materials_Tables_S1-S8_Figure_S1.pdf]

Supplementary Materials

Extreme Heat Exposure and Dementia-Related Mortality: A Systematic Review and Meta-Analysis

Table S1. PRISMA 2020 checklist
Table S2. Search strategies (Part A, original search; Part B, rerun and citation searching; Part C, supplementary PubMed search without the older-adult block)
Table S3. Adapted ROBINS-E: criteria applied, ratings, and rationale
Table S4. Mapping of populations and data sources and assessment of overlap
Table S5. Selected estimates, their sources, and application of the estimate-selection hierarchy
Table S6. Full-text reports excluded, with reasons
Table S7. Departures from the registered record (PROSPERO CRD420261436457)
Table S8. Numerically reported estimates for alternative heatwave definitions and age groups
Figure S1. Funnel plot of the six study-level estimates
File S1. R code for the meta-analyses (separate file: Supplementary_File_S1_analysis_code.R)
File S2. Study-level data (separate file: Supplementary_File_S2_study_data.csv)

Reference numbers in square brackets correspond to the reference list of the article.

Supplementary Table S1. PRISMA 2020 checklist

The checklist was completed against the revised manuscript. Locations refer to sections, tables, and figures of the revised manuscript and to these Supplementary Materials. Source of the checklist items: Page MJ, McKenzie JE, Bossuyt PM, et al. BMJ 2021;372:n71 (CC BY 4.0).

Section and topic	Item	Checklist item	Location where item is reported
**TITLE**			
Title	1	Identify the report as a systematic review.	Title
**ABSTRACT**			
Abstract	2	See the PRISMA 2020 for Abstracts checklist.	Abstract
**INTRODUCTION**			
Rationale	3	Describe the rationale for the review in the context of existing knowledge.	Introduction, paragraphs 1–3 (including the related review [47])
Objectives	4	Provide an explicit statement of the objective(s) or question(s) the review addresses.	Introduction, paragraph 4 (objective and a priori hypothesis); Methods 2.1
**METHODS**			
Eligibility criteria	5	Specify the inclusion and exclusion criteria for the review and how studies were grouped for the syntheses.	Methods 2.2 (criteria as registered and as applied); Methods 2.5; Methods 2.7
Information sources	6	Specify all databases, registers, websites, organisations, reference lists and other sources searched or consulted to identify studies. Specify the date when each source was last searched or consulted.	Methods 2.3 (original search, 15 June 2026; rerun and citation searching, 15 October 2026; supplementary PubMed search without the older-adult block, 22 October 2026); Table S2 (Parts A–C)
Search strategy	7	Present the full search strategies for all databases, registers and websites, including any filters and limits used.	Table S2 (Part A: original search, with a statement of which strategies were saved verbatim; Part B: rerun and citation searching; Part C: supplementary PubMed search)
Selection process	8	Specify the methods used to decide whether a study met the inclusion criteria of the review, including how many reviewers screened each record and each report retrieved, whether they worked independently, and if applicable, details of automation tools used in the process.	Methods 2.4 (two independent reviewers at every stage of the original search and the rerun; no automation tool was used for these). For the supplementary PubMed search, the records were pre-screened with the assistance of generative AI, and every screening and full-text decision was verified by the authors (Methods 2.4). Use of generative AI is described in Methods 2.3 and 2.4 and the Acknowledgments: it was not an information source, and every screened or included record was retrieved from the bibliographic databases or by citation searching.
Data collection process	9	Specify the methods used to collect data from reports, including how many reviewers collected data from each report, whether they worked independently, any processes for obtaining or confirming data from study investigators, and if applicable, details of automation tools used in the process.	Methods 2.5 (two independent reviewers; study authors were not contacted)
Data items	10a	List and define all outcomes for which data were sought. Specify whether all results that were compatible with each outcome domain in each study were sought, and if not, the methods used to decide which results to collect.	Methods 2.2 (Outcome); Methods 2.5 (estimate-selection hierarchy); Tables S5 and S8
Data items	10b	List and define all other variables for which data were sought. Describe any assumptions made about any missing or unclear information.	Methods 2.5; Tables 1 and 2
Study risk of bias	11	Specify the methods used to assess risk of bias in the included studies, including details of the	Methods 2.6 (adapted ROBINS-E assessment; rule for the overall judgment); Table

Section and topic	Item	Checklist item	Location where item is reported
assessment		tool(s) used, how many reviewers assessed each study and whether they worked independently, and if applicable, details of automation tools used in the process.	S3 (criteria; departure from the full ROBINS-E procedure is stated)
Effect measures	12	Specify for each outcome the effect measure(s) used in the synthesis or presentation of results.	Methods 2.7 (Effect measure); Table 2
Synthesis methods	13a	Describe the processes used to decide which studies were eligible for each synthesis.	Methods 2.5; Methods 2.7; Tables S4 and S5
Synthesis methods	13b	Describe any methods required to prepare the data for presentation or synthesis, such as handling of missing summary statistics, or data conversions.	Methods 2.7 (Effect measure): all included studies reported conditional odds ratios from time-stratified case-crossover analyses, which estimate rate ratios; they were pooled on the log scale without conversion to risk ratios, the standard error was derived from the reported 95% CI, and one estimate reported as a percentage change was re-expressed as a ratio (Table 2)
Synthesis methods	13c	Describe any methods used to tabulate or visually display results of individual studies and syntheses.	Methods 2.7; Figure 2; Tables 2, 4, and 5
Synthesis methods	13d	Describe any methods used to synthesize results and provide a rationale for the choice(s). If meta-analysis was performed, describe the model(s), method(s) to identify the presence and extent of statistical heterogeneity, and software package(s) used.	Methods 2.7 (Model); File S1
Synthesis methods	13e	Describe any methods used to explore possible causes of heterogeneity among study results.	Methods 2.7 (Exploratory stratification); planned subgroup analyses were not performed (Table S7)
Synthesis methods	13f	Describe any sensitivity analyses conducted to assess robustness of the synthesized results.	Methods 2.7 (Primary and sensitivity analyses)
Reporting bias assessment	14	Describe any methods used to assess risk of bias due to missing results in a synthesis.	Methods 2.7 (Small-study effects)
Certainty assessment	15	Describe any methods used to assess certainty (or confidence) in the body of evidence for an outcome.	Methods 2.8 (GRADE)
**RESULTS**			
Study selection	16a	Describe the results of the search and selection process, from the number of records identified in the search to the number of studies included in the review, ideally using a flow diagram.	Results 3.1; Figure 1 (original search, rerun, citation searching, and supplementary PubMed search); Table S2, Part C (screening decision for every record of the supplementary search)
Study selection	16b	Cite studies that might appear to meet the inclusion criteria, but which were excluded, and explain why they were excluded.	Results 3.1; Table S6 (each excluded full-text report with its reason)
Study characteristics	17	Cite each included study and present its characteristics.	Results 3.2; Tables 1 and 2
Risk of bias in studies	18	Present assessments of risk of bias for each included study.	Results 3.3; Table 3; Table S3 (rationale for each rating)
Results of individual studies	19	For all outcomes, present, for each study: (a) summary statistics for each group (where appropriate) and (b) an effect estimate and its precision, ideally using structured tables or plots.	Table 2; Figure 2; Table S8; File S2
Results of syntheses	20a	For each synthesis, briefly summarise the characteristics and risk of bias among contributing studies.	Results 3.2–3.3; Tables 1–3
Results of syntheses	20b	Present results of all statistical syntheses conducted. If meta-analysis was done, present for each the summary estimate and its precision and measures of statistical heterogeneity.	Results 3.4; Table 4; Figure 2
Results of syntheses	20c	Present results of all investigations of possible causes of heterogeneity among study results.	Results 3.6; Table 5; Table S8
Results of syntheses	20d	Present results of all sensitivity analyses conducted to assess the robustness of the synthesized results.	Results 3.4; Table 4 (all leave-one-out and sensitivity analyses, including the age-related analyses)
Reporting biases	21	Present assessments of risk of bias due to missing results (arising from reporting biases) for each	Results 3.5; Figure S1

Section and topic	Item	Checklist item	Location where item is reported
		synthesis assessed.	
Certainty of evidence	22	Present assessments of certainty (or confidence) in the body of evidence for each outcome assessed.	Results 3.7; Table 6
**DISCUSSION**			
Discussion	23a	Provide a general interpretation of the results in the context of other evidence.	Discussion (opening paragraph); Section 4.1
Discussion	23b	Discuss any limitations of the evidence included in the review.	Section 4.5
Discussion	23c	Discuss any limitations of the review processes used.	Section 4.5
Discussion	23d	Discuss implications of the results for practice, policy, and future research.	Section 4.4; Conclusions
**OTHER INFORMATION**			
Registration and protocol	24a	Provide registration information for the review, including register name and registration number, or state that the review was not registered.	Methods 2.1 (PROSPERO CRD420261436457; registered on 12 August 2026, after the searches had been run)
Registration and protocol	24b	Indicate where the review protocol can be accessed, or state that a protocol was not prepared.	Methods 2.1 (according to the PROSPERO record, the uploaded protocol will be made available after completion of the review; the registered record is publicly accessible); Table S7
Registration and protocol	24c	Describe and explain any amendments to information provided at registration or in the protocol.	Methods 2.1; Table S7
Support	25	Describe sources of financial or non-financial support for the review, and the role of the funders or sponsors in the review.	Funding statement
Competing interests	26	Declare any competing interests of review authors.	Conflicts of Interest statement
Availability of data, code and other materials	27	Report which of the following are publicly available and where they can be found: template data collection forms; data extracted from included studies; data used for all analyses; analytic code; any other materials used in the review.	Data Availability Statement; File S1 (analytic code) and File S2 (study-level data) are provided with the article; the data-extraction form is available from the corresponding author on request

Supplementary Table S2. Search strategies

Part A. Original search (database inception to 15 June 2026). Retrieval was limited to English-language records; no other filters were applied. A total of 389 records were retrieved before de-duplication. Only the PubMed strategy was saved verbatim at the time of the search. The strategies shown for the other three databases were reconstructed afterwards from the search record (search concepts and platform conventions) and may not reproduce the original record counts exactly; they were also not fully harmonized with the PubMed strategy (for example, several temperature terms are absent from the PubMed strategy, and "mental disorder*" is absent from the APA PsycINFO strategy). For this reason all searches were rerun (Part B).

Database	Status of the strategy	Search strategy	Period, limits, and records retrieved
PubMed	Saved verbatim	(("Dementia"[Mesh] OR "Dementia"[tw] OR "Alzheimer Disease"[Mesh] OR "Alzheimer*"[tw] OR "Cognitive Dysfunction"[Mesh] OR "Cognitive Decline"[tw] OR "Cognitive Impairment"[tw] OR "Mental Disorders"[Mesh])) AND (("Heat Wave*"[tw] OR "Heatwave*"[tw] OR "Extreme Heat"[tw] OR "Hot Temperature"[Mesh] OR "Hot Temperature*"[tw] OR "High Temperature*"[tw])) AND (("Aged"[Mesh] OR "Aged, 80 and over"[Mesh] OR "Elderly"[tw] OR "Older Adult*"[tw] OR "Older People"[tw] OR "Geriatric*"[tw]))	Inception to 15 June 2026; English; n = 225
Web of Science Core Collection	Reconstructed	TS=(dementia OR Alzheimer* OR "cognitive dysfunction" OR "cognitive decline" OR "cognitive impairment" OR "mental disorder*") AND TS=("heat wave*" OR heatwave* OR "extreme heat" OR "hot temperature*" OR "high temperature*" OR "ambient temperature*" OR "heat exposure" OR "temperature extreme*") AND TS=(aged OR elderly OR "older adult*" OR "older people" OR geriatric*)	Inception to 15 June 2026; English; field: Topic; n = 32
ProQuest Dissertations & Theses Global	Reconstructed	NOFT(dementia OR Alzheimer* OR "cognitive dysfunction" OR "cognitive decline" OR "cognitive impairment" OR "mental disorder*") AND NOFT("heat wave*" OR heatwave* OR "extreme heat" OR "hot temperature*" OR "high temperature*" OR "ambient temperature*" OR "heat exposure" OR "temperature extreme*") AND NOFT(aged OR elderly OR "older adult*" OR "older people" OR geriatric*)	Inception to 15 June 2026; English; field: anywhere except full text; n = 29
APA PsycINFO (EBSCOhost)	Reconstructed	S1: SU Dementia OR SU "Alzheimer's Disease" OR SU "Cognitive Impairment" OR TI (dementia OR Alzheimer* OR "cognitive dysfunction" OR "cognitive decline" OR "cognitive impairment") OR AB (dementia OR Alzheimer* OR "cognitive dysfunction" OR "cognitive decline" OR "cognitive impairment")<br>S2: TI ("heat wave*" OR heatwave* OR "extreme heat" OR "hot temperature*" OR "high temperature*" OR "ambient temperature*" OR "heat exposure" OR "temperature extreme*") OR AB ("heat wave*" OR heatwave* OR "extreme heat" OR "hot temperature*" OR "high temperature*" OR "ambient temperature*" OR "heat exposure" OR "temperature extreme*")<br>S3: SU Aged OR SU "Older Adults" OR TI (aged OR elderly OR "older adult*" OR "older people" OR geriatric*) OR AB (aged OR elderly OR "older adult*" OR "older people" OR geriatric*)<br>S4: S1 AND S2 AND S3	Inception to 15 June 2026; English; fields: subject headings, title, abstract; n = 103

Part B. Rerun with harmonized strategies.

Source	Search strategy	Period and records retrieved
PubMed	("Dementia"[Mesh] OR dementia[tw] OR "Alzheimer Disease"[Mesh] OR Alzheimer[tw] OR "Cognitive Dysfunction"[Mesh] OR "cognitive dysfunction"[tw] OR "cognitive decline"[tw] OR "cognitive impairment"[tw] OR "Mental Disorders"[Mesh] OR "mental disorder"[tw]) AND ("Extreme Heat"[Mesh] OR "Hot Temperature"[Mesh] OR "heat wave"[tw] OR heatwave[tw] OR "extreme heat"[tw] OR "hot temperature"[tw] OR "high temperature"[tw] OR "ambient temperature"[tw] OR "heat exposure"[tw] OR "temperature extreme"[tw]) AND ("Aged"[Mesh] OR "Aged, 80 and over"[Mesh] OR elderly[tw] OR "older adult"[tw] OR "older people"[tw] OR geriatric[tw]) AND English[la]	Inception to 15 Oct 2026; English; n = 237
Web of Science Core Collection	(dementia OR Alzheimer OR "cognitive dysfunction" OR "cognitive decline" OR "cognitive impairment") AND ("heat wave" OR heatwave OR "extreme heat" OR "hot temperature" OR "high temperature" OR "ambient temperature" OR "heat exposure" OR "temperature extreme") AND (aged OR elderly OR "older adult" OR "older people" OR geriatric)	Inception to 15 Oct 2026; English; n = 38
ProQuest Dissertations & Theses Global	Variant 1: (dementia OR Alzheimer OR "cognitive dysfunction" OR "cognitive decline" OR "cognitive impairment" OR "mental disorder") AND NOFT("heat wave" OR heatwave OR "extreme heat" OR "hot temperature" OR "high temperature" OR "ambient temperature" OR "heat exposure" OR "temperature extreme") AND NOFT(aged OR elderly OR "older adult" OR "older people" OR geriatric); language: English	Variant 1: Inception to 15 Oct 2026; English; n = 25<br>Variant 2: Inception to 15 Oct 2026;

Source	Search strategy	Period and records retrieved
	Variant 2: NOFT (dementia OR Alzheimer OR "cognitive dysfunction" OR "cognitive decline" OR "cognitive impairment" OR "mental disorder") AND NOFT("heat wave" OR heatwave OR "extreme heat" OR "hot temperature" OR "high temperature" OR "ambient temperature" OR "heat exposure" OR "temperature extreme") AND (aged OR elderly OR "older adult" OR "older people" OR geriatric); language: English	English; n = 9
EBSCOhost Complete Package	(dementia OR Alzheimer* OR "cognitive impairment" OR "cognitive decline" OR "neurocognitive disorder*") AND ("heat wave*" OR heatwave* OR "extreme heat" OR "high temperature*" OR "ambient temperature*" OR "heat exposure" OR "heat stress") AND (aged OR elderly OR "older adult*" OR "older people" OR geriatric*)	Inception to 15 Oct 2026; English; fields: subject headings, title, abstract; n = 118
Citation searching	Reference lists of the 5 included studies [49,52–55] and of the related review [47]; records citing the included studies (Web of Science)	Inception to 15 Oct 2026; English; n = 3

Handling of the rerun records: the exports from the four sources were merged in Microsoft Excel; duplicates within the rerun and records already retrieved in the original search were identified by DOI and by title, authors, and publication year and removed; the remaining records were screened on title and abstract, and potentially eligible records were assessed in full text, by two reviewers working independently against the same eligibility criteria as the original search. The number of records removed or excluded at each step was not documented for the rerun. No additional eligible study was identified. Citation searching identified three reports, which were assessed in full text (Table S6).

Note: Both searches combined three concept blocks (dementia, heatwaves or extreme heat, and older adults). Because the third block restricts retrieval to records that mention or are indexed for older adults, studies of dementia mortality that do not refer to age in the title, abstract, or indexing terms may not be retrieved; this is acknowledged as a limitation in Section 4.5 of the article.

Part C. Supplementary PubMed search without the older-adult block (second round of review). The search was run on 22 October 2026 through a PubMed search interface and was limited to records entered in PubMed up to 15 October 2026 (entry date), the date of the rerun. It used the dementia and heat blocks of the harmonized PubMed strategy (Part B) and the English-language limit, and it removed the records retrieved by the older-adult block (Boolean NOT), so that it returned only records that the older-adult block had excluded from the harmonized strategy. Because the interface accepts at most 20 Boolean operators per query, the dementia and heat blocks were each split into two halves and the search was run as four sub-queries, whose union is equivalent to the full query.

Query	Search string (PubMed; entry date up to 15 October 2026)	Records
Q1	("Dementia"[Mesh] OR dementia[tw] OR "Alzheimer Disease"[Mesh] OR Alzheimer[tw] OR "Cognitive Dysfunction"[Mesh]) AND ("Extreme Heat"[Mesh] OR "Hot Temperature"[Mesh] OR "heat wave"[tw] OR heatwave[tw] OR "extreme heat"[tw]) AND English[la] NOT ("Aged"[Mesh] OR "Aged, 80 and over"[Mesh] OR elderly[tw] OR "older adult"[tw] OR "older people"[tw] OR geriatric[tw])	172
Q2	("Dementia"[Mesh] OR dementia[tw] OR "Alzheimer Disease"[Mesh] OR Alzheimer[tw] OR "Cognitive Dysfunction"[Mesh]) AND ("hot temperature"[tw] OR "high temperature"[tw] OR "ambient temperature"[tw] OR "heat exposure"[tw] OR "temperature extreme"[tw]) AND English[la] NOT ("Aged"[Mesh] OR "Aged, 80 and over"[Mesh] OR elderly[tw] OR "older adult"[tw] OR "older people"[tw] OR geriatric[tw])	305
Q3	("cognitive dysfunction"[tw] OR "cognitive decline"[tw] OR "cognitive impairment"[tw] OR "Mental Disorders"[Mesh] OR "mental disorder"[tw]) AND ("Extreme Heat"[Mesh] OR "Hot Temperature"[Mesh] OR "heat wave"[tw] OR heatwave[tw] OR "extreme heat"[tw]) AND English[la] NOT ("Aged"[Mesh] OR "Aged, 80 and over"[Mesh] OR elderly[tw] OR "older adult"[tw] OR "older people"[tw] OR geriatric[tw])	647
Q4	("cognitive dysfunction"[tw] OR "cognitive decline"[tw] OR "cognitive impairment"[tw] OR "Mental Disorders"[Mesh] OR "mental disorder"[tw]) AND ("hot temperature"[tw] OR "high temperature"[tw] OR "ambient temperature"[tw] OR "heat exposure"[tw] OR "temperature extreme"[tw]) AND English[la] NOT ("Aged"[Mesh] OR "Aged, 80 and over"[Mesh] OR elderly[tw] OR "older adult"[tw] OR "older people"[tw] OR geriatric[tw])	840
Union	Q1 OR Q2 OR Q3 OR Q4 (records de-duplicated by PMID)	1,051
Step 1	Union AND (mortality[tw] OR death[tw] OR deaths[tw]), run as the four sub-queries above with this block added; records without these terms cannot report a mortality estimate and were excluded (n = 961)	90

The 90 records retained after step 1 were screened on title and abstract; their decisions are listed below. Five were reports already assessed in the review, 82 were excluded, and three were assessed in full text: one new study was included [51], one report was an additional report of an included study [54] that analyzed a subset of the same deaths (2016–2019) with the same F00–F09 definition [68], and one was excluded

(Table S6) [69]. Codes: NO, not an original study (review, systematic review, editorial, letter, news, commentary, protocol, case report or series, or projection); LAB, non-human or laboratory study; NM, no mortality outcome; NHE, no defined heatwave or extreme-heat episode; ND, no dementia-specific mortality estimate; AA, report already assessed in the review; FT, assessed in full text.

PMID	First author, year	Topic	Decision
42499040	Shao 2026	Global burden of injuries from fire, heat, and hot substances	ND
42489549	Tao 2026	Day and night heatwaves and mental disorder mortality, Shanghai	FT: additional report of an included study [68]
42485902	Friedman 2026	Disasters and drug overdose (framework)	NO
42101851	Srinivasan 2026	Heat vulnerability in autism and disability	NO
41544353	Li 2026	Extreme temperature events, air pollutants, and neurodegenerative disease mortality, Jiangsu	FT: included [51]
41390637	Yi 2025	Temperature and mortality in patients with severe mental disorders	ND
41125279	Chukwusa 2025	Extreme heat and mental health, London (protocol)	NO
41048198	Dennett 2025	Heat and drug overdose mortality	ND
40714605	Kirby 2025	Schizophrenia and extreme heat (review)	NO
40641135	Vrkljan 2025	Heat and psychiatric emergencies in pregnancy	NM
40490425	Nic Ionmhain 2025	Hyperthermia with illicit drugs (case reports)	NO
40037471	Iban-Arias 2025	Particulate matter and heat stress in a mouse model	LAB
39988049	Komulainen 2025	Childhood temperature and mental disorders	NM
39957242	Allison 2025	Psychiatrists and heat stress	NO
39888886	Tetzlaff 2025	Heat dome and toxic drug deaths (media analysis)	NO
38518609	Yin 2024	Extreme heat and cognitive function	NM
38150812	Meadows 2023	Mental illness and heat vulnerability (systematic review)	NO
38070557	Soravia 2023	Heat and cognition in wild birds	LAB
38055611	Jackson 2024	Extreme temperature, mortality, and admissions, East Sussex	ND (excess deaths only; no relative estimate)
38044672	Choo 2023	Heat-killed bacteria and amyloid-β in cell culture	LAB
37690069	Liu 2024	Extreme temperature events and dementia mortality, Jiangsu	AA [53]
37566416	Cloud 2023	Extreme heat and suicide watch in prisons	NM
37480464	Nozari 2023	Neuroprotection after head injury in heat (animal)	LAB
37257626	Li 2023	Climate and mental and behavioral health (systematic review)	NO
37107845	Woodland 2023	Climate and pre-existing mental illness (scoping review)	NO
36926982	Cornwall 2023	Schizophrenia and heat deaths (news)	NO
36779999	Minor 2023	Temperature and mental health, North Carolina	ND
36570623	Lin 2022	Neuroprotection in age-related macular degeneration (review)	NO
36245188	Ryus 2022	Opioid use disorder during a heat wave (case series)	NO
36115187	Komulainen 2022	Childhood climate and schizophrenia	NM
35933023	Kim 2022	Sex differences in COVID-19 (fever)	NHE
35627660	Thompson 2022	Heatwave mortality in England, 2020	AA [7]
33578223	Gulcebi 2021	Climate change and epilepsy (review)	NO
33297344	Stivanello 2020	Mental disorders and summer temperature mortality, Bologna	NHE (per 1 °C)
33069514	Zammit 2020	Neurological disorders and climate (review)	NO
32748466	Mewasingh 2020	Febrile seizures (review)	NO
32745780	Ma 2020	Cause-specific heat and cold burden, Jiangsu	AA [66]
32659069	Krawisz 2020	Climate destabilization and health (review)	NO

PMID	First author, year	Topic	Decision
32201298	Chen 2020	Heat and MDMA lethality in mice	LAB
31520832	Xu 2019	Heatwaves and post-discharge deaths from Alzheimer's disease, Brisbane	AA [55]
31230236	Ho 2019	Summer temperature and mental-disorder mortality, Hong Kong	NHE (per 1 °C)
30811527	Will 2019	N-acetylcysteine in heat-injury hepatitis (case report)	NO
30318181	Fais 2018	Fatal vehicular hyperthermia (case series)	NO
30286467	Wang 2018	Omi/HtrA2 in septic rats	LAB
30114719	Kovacs-Nagy 2018	HTRA2 defect (case reports)	NO
30021956	Lõhmus 2018	Mental health and heat (review)	NO
30007545	Thompson 2018	Heat and mental health outcomes (systematic review)	NO
29857067	Harrison 2018	Ethanol and burn injury (animal)	LAB
29768651	Nourbakhsh 2018	Cannabinoid hyperemesis deaths (case reports)	NO
29351426	Heinonen 2017	Heat, cold, and sauna (review)	NO
28651196	Nadesan 2017	Heat stroke at a festival (case report)	NO
28645059	Auger 2017	Temperature and cocaine overdose deaths	ND
28380698	Oliver 2017	KCNC1 myoclonus epilepsy (case series and in vitro)	NO
28343286	Liu 2017	Insect stings and heat exhaustion (case report)	NO
27720464	Krutmann 2016	Skin aging exposome (review)	NO
26965693	Goo 2017	HtrA2 in neurodegeneration (review)	NO
26896043	Ticehurst 2016	Heat waves and people with mental illness (letter)	NO
26502946	Merzetti 2015	Parkinson disease models in Drosophila	LAB
26001832	Lee 2015	Heat stress and memory in mice	LAB
25692296	Kim 2015	Heat-attributable deaths, Seoul	FT: excluded [69]
25317814	Liu 2014	Nicotine withdrawal and pain in rats	LAB
23016688	Skorko-Glonek 2013	HtrA proteases as drug targets (review)	NO
22401992	Drouin-Ouellet 2012	MyD88-deficient mice	LAB
21214621	Cusack 2011	Heatwaves, substance use, and mental health (discussion paper)	NO
20219056	Bohnert 2010	Temperature and overdose deaths, New York City	ND
20033251	Berry 2009	Climate and mental health (framework)	NO
19530165	King 2009	Hsp27 and amyloid-β in neurons	LAB
19241651	Venables 2007	Prenatal influenza and delivery complications	NM
19007525	Shakleya 2008	Smoked methamphetamine marker (case report)	NO
18941580	Hansen 2008	Heat waves and mental health, Adelaide	AA [50]
18662332	Inagaki 2008	Omi/HtrA2 in Huntington disease	LAB
18098216	Flanagan 2008	Blood toxicity of psychotropic drugs (review)	NO
18077561	Muresanu 2007	Hypertension and heat stress in rats	LAB
17209275	Woodruff 2006	Health projections of climate policy	NO
16051598	Fujimoto 2005	HSF1 and polyglutamine	LAB
15294909	Gupta 2004	Presenilin and Omi/HtrA2	LAB
12590048	Moriya 2003	Nitrazepam intoxication (case report)	NO
12350296	Kahn 2002	Physiology of sudden infant death (review)	NO

PMID	First author, year	Topic	Decision
12168692	Jackson 2002	Aged garlic and caspase-3	LAB
12019670	Leung 2002	Charcoal-burning suicide, Hong Kong	ND
9987864	Moriya 1999	Postmortem drug redistribution (case reports)	NO
9865243	Ramcharan 1998	Survival after ecstasy overdose (case report)	NO
9712220	Bark 1998	Deaths of psychiatric patients in heat waves, New York City	ND
9628717	Kilbourne 1998	Cocaine use and heat-wave deaths (editorial)	NO
9628710	Marzuk 1998	Temperature and cocaine overdose mortality	ND
8719620	Feng 1995	Sleep deprivation in lesioned rats	LAB
8108423	Abrahamson 1994	Cystatin C aggregation and temperature	LAB
4040123	Chan 1985	Ethanol, heat stress, and hypertension in rats	LAB
2373059	Balzano 1990	Sleep deprivation and brown-fat deiodinase in rats	LAB
1866525	Prete 1991	Sleep deprivation and temperature preference in rats	LAB

Supplementary Table S3. Adapted ROBINS-E: criteria applied, ratings, and rationale

Part A. Criteria. Domain-level ratings were reached with the review-specific criteria below, which operationalize each ROBINS-E domain [56] for studies of heat and dementia-related mortality. The full set of ROBINS-E signaling questions and the associated algorithms were not completed; the assessment is therefore an adapted ROBINS-E assessment, and its judgments are not equivalent to those of a complete assessment with the instrument (Sections 2.6 and 4.5 of the article). The overall judgment was derived from the domain-level judgments as shown in the last row. Criteria that referred to outcomes outside the scope of the review (hospitalization and emergency admission) have been removed from the outcome-measurement domain.

Domain	Low risk of bias	Some concerns	High risk of bias
D1. Confounding	Design and analysis control for the major confounders of the short-term association between heat and mortality (temporal patterns, season, day of the week, humidity, air pollution where available) and for relevant individual and contextual characteristics.	Several key confounders are addressed, but potentially important factors—indoor temperature, access to air conditioning, medication use, dementia severity, residential environment, or individual adaptive capacity—are not accounted for.	Major temporal, environmental, demographic, or clinical confounders are not adequately controlled.
D2. Measurement of the exposure	The heatwave or extreme-heat exposure is defined reproducibly (threshold, duration, lag, intensity) and is assigned from reliable data that capture individual exposure.	The exposure is clearly defined but relies on outdoor ambient temperature at city, regional, area, grid, or residential-location level, which may not reflect individual exposure, indoor temperature, cooling access, or time spent indoors.	The exposure definition is unclear, is not temporally linked to the outcome, or uses crude or inappropriate indicators.
D3. Selection of participants	Cases are identified from population-based, official, or near-complete sources with clearly defined eligibility.	The population comes from a valid but geographically limited, facility-based, single-city, or otherwise restricted source that raises concerns about representativeness.	Selection is likely to be related to both heat exposure and the outcome, eligibility is unclear, or highly selected cases are included without justification.
D4. Post-exposure interventions	Post-exposure interventions or adaptive responses are unlikely to differ systematically between exposed and unexposed periods, or the design minimizes their influence.	Heat alerts, cooling-center access, air-conditioning use, institutional heat-prevention measures, caregiver responses, medication adjustment, or other adaptive behaviors may have influenced the association but were not measured or adjusted for.	Post-exposure interventions or avoidance behaviors are likely to differ substantially across exposure conditions and may seriously distort the estimate.
D5. Missing data	Outcome, exposure, and covariate data are nearly complete, come from reliable official sources, and missingness is minimal or appropriately handled.	Missing data are incompletely described, some cases are excluded because of missing address, exposure, covariate, or outcome information, or the handling of missing data is only partly reported.	A substantial proportion of data is missing, missingness is likely to be related to exposure and outcome, or missing data are not addressed.
D6. Measurement of the outcome	Dementia-related mortality is measured from official death registries or linked administrative records using standardized diagnostic codes.	The outcome definition is generally appropriate but broad—for example, dementia presented as a subgroup of total mental disorders or extracted from a wider diagnostic category.	The outcome is poorly defined, based on non-standard criteria, or cannot be distinguished from unrelated mental or neurological outcomes.
D7. Selection of the reported result	Heatwave definitions, thresholds, lags, models, subgroup analyses, and sensitivity analyses are clearly reported, and the main findings are presented transparently.	Multiple exposure definitions, lags, subgroups, or models are examined, but it is unclear whether all results are fully reported, or no prespecified analysis plan is available.	The study appears to report selectively the statistically significant definitions, lags, subgroups, or outcomes, or the reported estimate appears to have been selected from multiple analyses without sufficient transparency.
Overall (derived from the domain-level judgments; the overall judgment is at least as severe as the most severe domain-level judgment)	All domains at low risk.	At least one domain raises some concerns and no domain is at high or very high risk.	At least one domain at high risk. Very high risk: at least one domain at very high risk, or several domains at high risk.

Part B. Ratings after re-assessment, with rationale. The initial ratings were re-assessed against the criteria in Part A during revision; ratings that changed are marked with a dagger (†). The study identified in the second round of review [51] was rated with the same criteria. L, low risk of bias; SC, some concerns; H, high risk of bias.

Study	D1 Confounding	D2 Exposure	D3 Selection	D4 Post-exposure interventions	D5 Missing data	D6 Outcome	D7 Reported result	Over all
Gao et al. (2024) [52]	SC. Case-crossover design controls time-invariant factors, trend, and season; humidity and holidays adjusted; air pollution only in a sensitivity analysis; indoor conditions, air conditioning, and medication not measured.	SC. Reanalysis temperature assigned at county level; outdoor exposure only.	L. National reporting system covering all county-level districts of mainland China.	SC†. Adaptive behavior, cooling, and heat-warning responses not measured.	SC†. Exclusions and missing records not described in the article.	L. Dementia (F00–F03, G30, G31) as the underlying cause in the national death registration system.	SC. Four thresholds and two metrics examined; no prespecified primary definition.	SC
Li et al. (2026) [51]	SC. Case-crossover design; air quality index and relative humidity adjusted; indoor conditions, air conditioning, and medication not measured.	SC. ERA5-Land reanalysis temperature (0.1°) assigned to the residential address; outdoor exposure only.	L. Province-wide mortality surveillance system (population of about 85 million).	SC. Not measured.	SC. Exclusions and missing records not described in the article.	L. Dementia (Alzheimer's disease and non-Alzheimer dementias) coded according to ICD-10 in the provincial mortality surveillance system; codes listed in the supplement of the article.	SC. Fifteen heatwave definitions examined; the reported definition was selected by the Akaike information criterion, and estimates for the other definitions were shown only graphically.	SC
Liu et al. (2024) [53]	SC†. Case-crossover design; humidity adjusted; air pollution not in the main model; indoor conditions, air conditioning, and medication not measured.	SC†. Gridded outdoor temperature at the residential address; indoor exposure not captured.	L. Province-wide mortality surveillance system (population of about 85 million).	SC†. Not measured.	L. Selection of deaths documented in the article's supplementary flow chart; negligible missing covariate data.	L. Dementia (F01, F03, G30, G31) as the underlying cause of death.	SC†. Twelve heatwave definitions examined; no prespecified primary definition.	SC†
Tao et al. (2025) [54]	SC. Case-crossover design; humidity adjusted; air pollution not adjusted; indoor conditions and medication not measured.	SC. City-wide average of reanalysis temperature; no individual or district-level assignment.	SC†. Single city.	SC†. Not measured.	SC†. Exclusions and missing records not described.	SC. Deaths coded F00–F09 labeled as dementia; underlying versus contributing cause not stated; dementia analyzed within mental disorders.	SC. Nine definitions examined; dementia-specific estimates given numerically for one definition only; no prespecified primary definition.	SC
Xu et al. (2019) [55]	SC. Case-crossover design; PM_{10}, NO_2, and humidity adjusted; ozone and $PM_{2.5}$ unavailable; indoor conditions and medication not measured.	SC. City-wide series from two monitoring stations.	SC†. Single city; cohort restricted to patients hospitalized for Alzheimer's disease who died within two months of discharge; in-hospital deaths excluded.	SC†. Not measured (post-discharge care setting unknown).	SC. Handling of missing data not reported.	L. Alzheimer's disease as the underlying cause in a death registry linked to hospital records.	SC. Three definitions and eight lags examined; a numerical estimate was given only for the definition and lag with a statistically significant result, the others being shown graphically.	SC
Zhang et al. (2024) [49]	SC. Case-crossover design; humidity, pressure, and holidays adjusted; air pollution not adjusted; indoor conditions and medication not measured.	SC†. Station data interpolated to the residential location; outdoor exposure only.	L. National reporting system; restricted to ages ≥60 years.	SC†. Not measured.	SC†. Exclusions not described; the authors note possible reporting omissions in the system.	L. Dementia (F01, F03, G30, G31) from the national death registration system.	SC. Nine heatwave scenarios examined; no prespecified primary definition.	SC

Supplementary Table S4. Mapping of populations and data sources of the included studies and assessment of overlap

The country and region, study years, mortality data source, and age range of each included study were mapped to identify populations contributing to more than one estimate.

Study	Country and region	Study years (months analyzed)	Mortality data source	Age range	Dementia-related deaths (n)	Assessment of overlap	Handling in the synthesis
Gao et al. (2024) [52]	China; all 2,844 county-level districts of mainland China	2013–2019 (May–September)	China Cause of Death Reporting System (CDRS)	All ages (81.6% aged ≥75 years)	132,573	Same national reporting system as [49] for overlapping years (2013–2019). Deaths at ≥60 years occurring in May–September 2013–2019 are expected to be largely contained in the dataset of [49]; the exact extent of overlap cannot be determined from the published reports. The two estimates are not independent.	Not pooled in the primary analysis. Substituted for [49] in a sensitivity analysis.
Li et al. (2026) [51]	China; Jiangsu Province	2014–2021 (all months; heatwaves defined for May–September)	Mortality Surveillance System of the Jiangsu Provincial Center for Disease Control and Prevention	Adults (72.5% of dementia deaths at >80 years)	64,226	Same provincial surveillance system as [53] for overlapping years (2015–2020); the dementia deaths of [53] are expected to be largely contained in this dataset, and the two estimates are not independent. Deaths in Jiangsu may also be counted in [49]: even if all were, they would make up at most 16.1% of the deaths in [49].	Pooled in the primary analysis (longer study period and larger number of deaths than [53]). Possible dependence on [49] examined in a multivariate model with assumed correlations of 0–0.8.
Liu et al. (2024) [53]	China; Jiangsu Province	2015–2020 (all months)	Jiangsu provincial mortality surveillance system	≥18 years (mean age 84.2 years, SD 9.2; 53.5% aged ≥85 years)	57,791	Same provincial surveillance system as [51] for overlapping years. The national system receives death reports from all provinces, so dementia deaths in Jiangsu may also be counted in [49] (and, for May–September 2015–2019, in [52]).	Not pooled in the primary analysis (shorter study period and fewer deaths than [51]). Substituted for [51] in a sensitivity analysis.
Tao et al. (2025) [54]	China; Shanghai	2008–2021 (May–September)	Shanghai Municipal Center for Disease Control and Prevention mortality surveillance data	All ages (age distribution of dementia deaths not reported)	3,322	Deaths in Shanghai during 2013–2020 may also be counted in [49] (and, for 2013–2019, in [52]). A later report from the same research group and data source analyzed 931 deaths coded F00–F09 in 2016–2019, a subset of these deaths, and was treated as an additional report of this study [68].	Not pooled in the primary analysis (outcome F00–F09; heatwave effect conditional on temperature). Indirect evidence, added in sensitivity analyses.
Xu et al. (2019) [55]	Australia; Brisbane, Queensland	Admissions 2005–2013; deaths to February 2014 (all months)	Queensland Hospital Admitted Patient Data Collection linked to the Registrar General Death Database	Patients hospitalized for Alzheimer's disease (98.4% of decedents aged ≥65 years)	307	No overlap with any other included study.	Pooled in the primary analysis (the only cohort of people with a recorded diagnosis). Omitted in the leave-one-out analysis (outcome-definition sensitivity analysis).
Zhang et al. (2024) [49]	China; nationwide	2013–2020 (all months)	China Cause of Death Reporting System (CDRS); a companion report describes the 605 disease surveillance points as its core data source [34]	≥60 years (85.5% aged ≥75 years)	399,036	Overlaps with [52] (same system, overlapping years). Likely partial overlap with [53], [54], and [51]. A further report from the same dataset [34] was excluded at full-text assessment as an overlapping dataset.	Pooled in the primary analysis (restricted to ≥60 years; longer study period and larger number of deaths than [52]).

Abbreviations: CDRS, China Cause of Death Reporting System; ICD-10, International Classification of Diseases, 10th Revision; SD, standard deviation.

Supplementary Table S5. Selected estimates, their sources, and application of the estimate-selection hierarchy

Hierarchy (Section 2.5): (1) all dementia deaths in the whole study population rather than a subgroup; (2) percentile-based definition with the threshold closest to the 95th percentile, or the absolute threshold when no percentile-based definition was reported; (3) minimum duration of three consecutive days when reported numerically for that duration, otherwise two days; (4) definition based on daily mean temperature, or the daytime metric when only daytime and nighttime metrics were reported; (5) cumulative lag period and main model designated by the original authors; (6) estimates given as numbers in the text, tables, or figure labels rather than read from plotted points. The hierarchy was formalized post hoc during peer review.

Study	Estimates reported	Estimate selected by the hierarchy (95% CI)	Location in the original publication	Rules that determined the selection	Estimate used in the initial analysis (95% CI)	Changed
Gao et al. (2024) [52]	Hot-night excess (HNE) and hot-day excess (HDE), each with hot days or nights defined by the 90th, 92.5th, 95th, and 97.5th percentiles; overall and by dementia subtype, age, sex, education, and region; sensitivity models	Extreme HDE, 95th-percentile definition, overall: OR 1.62 (1.43–1.83); cumulative lag 0–13 d	Table 1 (also Table 2, "Overall")	Rule 1 (overall); rule 2 (95th percentile); rule 3 not applicable (no duration criterion); rule 4 (daytime metric; no estimate for a definition based on daily mean temperature was reported); rule 5 (cumulative lag, main model). Not selected: HNE, 95th percentile, 1.46 (1.30–1.63).	1.62 (1.43–1.83)	No
Li et al. (2026) [51]	Heatwave definitions combining the 90th, 92.5th, 95th, 97.5th, and 99th percentiles with ≥2, ≥3, and ≥4 d; the definition with the lowest Akaike information criterion (92.5th percentile, ≥4 d) used for all reported results; total neurodegenerative disease, dementia, Alzheimer's disease, non-Alzheimer dementias, and Parkinson's disease; single-day and cumulative lags 0–14 d	92.5th percentile, ≥4 d, dementia: OR 1.82 (1.61–2.06); cumulative lag 0–14 d	Results text (Section 3.2)	Rule 1 (all dementia); rule 6 (only definition reported numerically; others shown graphically in the supplement of the article); rule 5 (cumulative lag). Used in a sensitivity analysis: Alzheimer's disease deaths, 1.97 (1.59–2.44).	Not applicable (identified in the second round of review)	—
Liu et al. (2024) [53]	Twelve heatwave definitions (90th, 92.5th, 95th, and 97.5th percentiles × ≥2, ≥3, and ≥4 d); all dementia and Alzheimer's disease; cumulative and added-effect models; strata by sex and age	95th percentile, ≥3 d, all dementia: OR 1.75 (1.61–1.90); cumulative lag 0–10 d	Table 2	Rule 1; rule 2 (95th percentile); rule 3 (≥3 d); rule 4 (daily mean); rule 5 (cumulative lag, main model rather than the added-effect model).	1.750 (1.611–1.901)	No
Tao et al. (2025) [54]	Nine definitions (90th, 95th, and 99th percentiles × ≥2, ≥3, and ≥4 d), with 90p_2d, 95p_3d, and 99p_4d presented as main results; intensity-related (main) and added effects; dementia-specific estimates given numerically only for 90p_2d	90th percentile, ≥2 d, added effect: OR 1.13 (0.95–1.34); same-day exposure	Results text (dementia-specific estimates for other definitions are shown only graphically in Figure 2C)	Rule 6 (only numerically reported dementia-specific definition); comparator rule in Section 2.2 (added effect = heatwave days vs. non-heatwave days). Used in a sensitivity analysis: intensity-related effect 1.51 (0.94–2.43).	1.130 (0.951–1.342)	No
Xu et al. (2019) [55]	Three definitions (90th, 95th, and 97th percentiles, each for ≥2 d); lags 0–7 d; hospitalization and post-discharge death; a numerical estimate for post-discharge death given only for the 95th percentile	95th percentile, ≥2 d, post-discharge death: OR 3.69 (1.76–7.65), reported as an increase of 269% (76–665%); same-day exposure	Abstract (the lag is not stated with the number; the same-day estimate plotted in Figure 2 corresponds to it)	Rule 2 (95th percentile); rule 3 (only ≥2 d examined); rule 4 (daily mean); rule 6 (only numerically reported estimate). This is why a study that examined three thresholds is classified under the 95th percentile.	3.690 (1.770–7.693)	No (CI limits now as reported by the original authors)
Zhang et al. (2024) [49]	Nine heatwave scenarios (90th, 95th, and 97.5th percentiles × ≥2, ≥3, and ≥4 d), all given as	95th percentile, ≥3 d: cumulative OR 1.268 (1.236–1.300); cumulative	Figure 2b (numeric label)	Rule 2 (95th percentile); rule 3 (≥3 d reported numerically); rule 5 (cumulative lag 0–7 d); rule 6. Used	90th percentile & 2 d: 1.140 (1.118–1.163)	Yes

Study	Estimates reported	Estimate selected by the hierarchy (95% CI)	Location in the original publication	Rules that determined the selection	Estimate used in the initial analysis (95% CI)	Changed
	numeric labels in Figure 2 of the article; strata by sex, age, and region	lag 0–7 d		in sensitivity analyses: 95th percentile & 2 d, 1.250 (1.220–1.281) (Figure 2b; also given in the text of [34]); 90th percentile & 2 d, 1.140 (1.118–1.163).		

Abbreviations: CI, confidence interval; d, consecutive days; OR, odds ratio.

Supplementary Table S6. Full-text reports excluded, with reasons (n = 15)

Report	Design and setting	Primary reason for exclusion	Explanation
**Reports identified in the original database search (n = 12)**			
Farugia et al. (2021) [11]	Narrative review of extreme heat, bushfires, and dementia	Not an original study	Review article; no original effect estimate.
Eggenberger et al. (2021) [13]	Cross-sectional study of body temperature and cognitive performance in older adults with and without mild cognitive impairment	Ineligible population, exposure, and outcome	No ambient heat exposure, no dementia population, and no mortality outcome.
Hansen et al. (2008) [50]	Ecological time series of heatwaves and mortality from mental and behavioral disorders, Adelaide, Australia (1993–2004)	Estimate does not concern older adults (excluded during revision)	A dementia mortality estimate (incidence rate ratio 5.06, 95% CI 1.21–21.23) was reported only for people aged 15–64 years, and no all-age or older-adult estimate was given. The study was included in the initial submission and was excluded during revision because it provides no evidence on the target population (Section 2.2; Table S7).
Zanobetti et al. (2013) [5]	Case-only analysis of deaths among Medicare beneficiaries in 135 cities, United States	Incompatible estimand (effect modification)	Previous admission for Alzheimer's disease or dementia was examined as a modifier of mortality on extremely hot days (additional increase in risk of 8% [95% CI 4–12%] and 6% [3–9%], respectively); the heat effect on mortality among people with dementia was not estimated. Considered narratively in Section 4.1.
Foroni et al. (2007) [6]	Retrospective case–control study of older people who died or survived during August 2003, Modena, Italy	Incompatible estimand	Risk factors for death during a single heatwave (including cognitive impairment) were compared between decedents and survivors; there was no contrast between heatwave and non-heatwave periods.
Fritze (2020) [46]	Cohort of German health-claims data (≥65 years, 2004–2010); Cox models with dementia × temperature interaction	Incompatible estimand (interaction)	Exposure was at least one day with a Heat Index ≥27 °C in the preceding 0–3 days rather than a heatwave event; results were a main effect among people without dementia (hazard ratio 0.98, p = 0.521) and an interaction with dementia (1.11, p = 0.011). No confidence interval was reported, and the heat effect among people with dementia with its uncertainty could not be derived. Considered narratively in Section 4.1.
Page et al. (2012) [35]	Time-series analysis in a primary-care cohort of patients with psychosis, dementia, or substance misuse, England	Ineligible exposure contrast	Reported the change in mortality per 1 °C above a temperature threshold; per-degree estimates were excluded by the eligibility criteria (Section 2.2). Considered narratively in Section 4.1.
Astone and Vaalavuo (2022) [42]	Register-based municipality-month panel study of high temperatures, hospital visits, and mortality, Finland	Incompatible estimand	Estimated the percentage change in monthly mortality per additional day above 25 °C, including among older people with pre-existing dementia; not a contrast between heatwave and non-heatwave periods.
Thompson et al. (2022) [7]	Observational study of heatwave mortality in England, summer 2020	Effect measure not extractable	Reported absolute excess deaths (observed minus expected) from Alzheimer's disease and dementia during three heatwaves; no relative effect estimate with a measure of uncertainty.
Conte Keivabu et al. (2025) [45]	German health-claims data (≥50 years, 2004–2019); individual fixed-effects linear probability models	Effect measure not extractable	Reported changes in the probability of death in percentage points per additional day in a temperature band, stratified by dementia; not a ratio measure for a heatwave event. Considered narratively in Section 4.1.
Conti et al. (2007) [70]	Mortality among people older than 74 years during the 2003 heatwave, Genoa, Italy	Effect measure not extractable	Compared observed with expected deaths and the distribution of causes of death in 2003 and 2002; no dementia-specific relative estimate with a measure of uncertainty.
Zhang et al. (2026) [34]	Projection of dementia deaths attributable to extreme temperature events, China	Overlapping dataset	The exposure–response estimate is derived from the same 399,036 deaths (2013–2020, ≥60 years) as the included study [49] and restates its estimate.

Report	Design and setting	Primary reason for exclusion	Explanation
**Reports identified by citation searching, 15 October 2026 (n = 2)**			
Ma et al. (2020) [66]	Multicity time-series study of heat, cold, and cause-specific mortality in 11 cities of Jiangsu Province, China (2014–2017); identified from the reference list of [52]	Ineligible exposure contrast	Distributed lag non-linear models; reported the relative risk of death from Alzheimer's disease and dementia (ICD-10 F00–F03, G30–G31.1, G31.8–G31.9) at the 97.5th percentile of daily mean temperature relative to the minimum-mortality temperature (relative risk 1.99, 95% CI 1.26–3.14), together with the attributable fraction. This is a contrast between two points of a continuous temperature–mortality function, not an estimate for a defined heatwave or extreme-heat episode (Section 2.2). The deaths also overlap in place and period with those of the included Jiangsu study [53].
Lee et al. (2018) [67]	Two-stage time-series study of heatwaves, cold spells, and mortality in 53 communities in Korea and Japan (1992–2015); identified from the reference list of [54]	Ineligible outcome	Reported heatwave-related mortality for the total population by decade; no estimate for dementia-related mortality or for people with dementia.
**Report identified by the supplementary PubMed search, 22 October 2026 (n = 1)**			
Kim et al. (2015) [69]	Time-series study of heat and cause-specific mortality, Seoul, South Korea (1992–2009)	Ineligible exposure contrast and outcome	Reported the relative risk per 1 °C increase in daily maximum temperature above the 90th-percentile threshold, which the eligibility criteria exclude (Section 2.2); dementia was not reported separately (the narrowest category was ICD-10 F00–F09).

Note: Citation searching identified three reports, one from the reference list of each of three included studies [52–54]; the report identified from the reference list of [53] was an included study [55] and is therefore not listed. Of the three reports assessed in full text after the supplementary PubMed search, one was included [51] and one was an additional report of an included study [54,68]; neither is listed. The rerun of the database searches on 15 October 2026 identified no additional eligible study (Table S2, Part B). Twelve records were excluded at the title and abstract stage of the original search as not relevant to the review question. Morita et al. (2026) [33], a cohort study of extreme heat and the onset of dementia among people who were free of dementia at baseline, did not concern mortality among people with dementia and was not assessed in full text.

Supplementary Table S7. Departures from the registered record (PROSPERO CRD420261436457)

The review was registered on 12 August 2026, after the database searches had been run (15 June 2026) and while record screening was under way. The registered record therefore already reflected decisions taken with knowledge of the candidate studies. Entries in the column “Registered record” are taken from the PROSPERO record (version 1.0, published 12 August 2026).

Item	Registered record	What was done	Reason	Consequence
Timing of registration	First submitted and registered on 12 August 2026; review start date 15 June 2026. At submission, formal searching was recorded as completed and screening as started.	Registered after the searches and during screening; the initial submission described the registration as prospective.	The record was submitted late. The description has been corrected (Section 2.1).	The review does not have the protection against selective decisions that prospective registration provides.
Protocol availability	A full protocol was written and uploaded to PROSPERO and will be made available after the review is completed.	The initial submission described the protocol as publicly accessible; this was corrected (Section 2.1).	The protocol is not yet publicly available.	The registered record is the publicly accessible statement of the planned methods; departures are assessed against it in this table.
Age criterion	Adults aged ≥60 years with diagnosed dementia; population-based studies that include some participants younger than 60 years may be retained as age-indirect evidence when age-specific estimates are unavailable. An earlier, unregistered working plan specified ≥65 years.	As registered; the initial submission described the change from 65 to 60 years as occurring during data extraction.	The 60-year cut-off and the age-indirect clause were adopted after candidate studies had been identified. Both are post hoc.	Only one study is restricted to ≥60 years.
Studies with estimates only for ages <65 years	Not addressed explicitly; the target population was adults aged ≥60 years.	Hansen et al. [50], which was included in the initial submission, was excluded from the review.	Its dementia mortality estimate applied only to people aged 15–64 years and therefore provided no evidence on older adults. Decided during peer review, with knowledge of the initial results. In the second round of review, the criterion was reformulated by the age range to which an estimate refers (Section 2.2).	One study fewer; the primary analysis set at that time was unaffected.
Dementia definition	Diagnosed dementia ascertained by a clearly described method; eligible categories were Alzheimer's disease, vascular dementia, other specified dementias, and unspecified dementia (no diagnostic codes were listed; these categories correspond to ICD-10 F00–F03 and G30). Studies in which dementia was not separately identifiable from broader neurological or psychiatric conditions were to be excluded.	Definitions including G31 (three studies) and the block F00–F09 (one study [54]) were accepted.	Definitions were taken as specified by the original authors; retaining [54] departs from the registered exclusion criterion.	Outcome indirectness; sensitivity analysis excluding the F00–F09 study; an analysis restricted to matching definitions was not feasible.
Information sources	Main sources PsycINFO and PubMed; other sources Web of Science Core Collection and ProQuest; dissertation and thesis databases; English only; no date restriction. Citation searching was not listed.	Original search as registered. In the rerun on 15 October 2026, the EBSCOhost Complete Package was searched in place of APA PsycINFO alone, and citation searching of the included studies and of a related review [47] was added (Table S2, Part B). In the second round of review, a PubMed search without the older-adult block was added (Table S2, Part C).	Added in response to peer review to make the search reproducible and to address the sensitivity of the older-adult block.	The rerun and citation searching identified no additional eligible study; the supplementary PubMed search identified one (Li et al. [51]), which entered the primary analysis.
Search for unpublished	Both published and unpublished studies to be	One dissertation database (ProQuest Dissertations & Theses	Resource constraints.	Unpublished studies may have been

Item	Registered record	What was done	Reason	Consequence
studies	sought, including dissertation and thesis databases.	Global) was searched; no other gray-literature source.		missed.
Screening sequence	Records to be de-duplicated in Microsoft Excel, followed by title-and-abstract screening and full-text assessment by two independent reviewers.	Titles were first screened within each database export, and duplicates were removed afterwards; title-and-abstract screening and full-text assessment then followed as registered.	Practical handling of the database exports.	The number of unique records retrieved in the original search is not available (Section 2.4; Figure 1).
Contact with study authors and investigators	Authors to be asked for data not available in published reports; investigators to be contacted when information needed for the risk-of-bias assessment was unclear or unavailable.	Not done.	Not undertaken within the review timeline.	The numerical estimates shown only graphically in [54] and [55] were not obtained; risk of bias was rated from the published reports.
Effect measure	Hazard ratio with 95% CI as the primary effect measure; the compatibility of other relative measures to be assessed, and only sufficiently comparable estimates pooled.	No study reported hazard ratios; all included studies reported odds ratios from conditional logistic regression in case-crossover analyses, which were pooled without conversion and are presented as odds ratios.	Corrected in response to peer review.	Labeling and interpretation changed; numerical inputs unchanged.
Estimate selection	Where multiple adjusted estimates were available, the estimate "most directly aligned with the review question and based on the most appropriate adjusted model".	Explicit six-rule hierarchy (Section 2.5; Table S5).	Formalized post hoc in response to peer review.	The estimate for one study [49] changed.
Independence of estimates	Not addressed in the record.	Initially two studies of the same national system were pooled; one study from each pair based on the same data source (national and Jiangsu provincial) is now used in sensitivity analyses only, and residual dependence between the national and provincial datasets was examined in a multivariate model.	Overlap identified during peer review.	Three studies are pooled in the primary analysis; residual dependence had a negligible influence on the pooled estimate.
Primary analysis set (second round of review)	Not addressed in the record (all eligible studies to be pooled when sufficiently comparable).	Restricted to estimates with a dementia-specific outcome definition and an estimate of the total heatwave association; the study using ICD-10 F00–F09 and an effect conditional on temperature [54] is presented as indirect evidence.	Requested in peer review to improve the comparability of the pooled estimates; decided with knowledge of the previous results.	Primary analysis of three studies (OR 1.84, 95% CI 0.54–6.25); the four-study analysis of the first revision is reported as a sensitivity analysis.
Statistical model and software	Random-effects model (estimator not specified); Cochran's Q, I^2, and a 95% prediction interval; leave-one-out analysis; Comprehensive Meta-Analysis version 4; two-sided $p < 0.05$.	Initially DerSimonian–Laird with a Wald-type CI in Comprehensive Meta-Analysis; now REML with the Hartung–Knapp CI in R (metafor). Q, I^2, the prediction interval, and the leave-one-out analysis are reported as registered.	Changed in response to peer review, with knowledge of the initial results.	The pooled CI is wider and includes the null.
Subgroup analyses and meta-regression	By country or geographic setting, heat-exposure threshold, and heatwave duration where sufficient data are available; meta-regression only with ≥10 studies.	Country and threshold were initially analyzed as subgroups, and duration was not analyzed; all three are now presented descriptively without tests. No meta-regression was performed.	Too few studies per category; characteristics confounded.	No inference about subgroup differences.
Age-related sensitivity analysis	Not registered (the registered sensitivity analysis was leave-one-out only).	Added (Table 4).	Needed to address age indirectness.	—
Trim-and-fill	Not registered (visual inspection of funnel plots; formal asymmetry tests only with ≥10 studies).	Run in the initial analysis and shown in the funnel plot without being described; now withdrawn and reported for transparency only (Section 3.5).	Software default in the initial analysis.	No inference is drawn from it.
Risk of bias	ROBINS-E, assessed independently by two	Review-specific criteria operationalizing the ROBINS-E	Initial ratings were more favorable than	No study is rated as low risk overall.

Item	Registered record	What was done	Reason	Consequence
	reviewers.	domains; signaling questions not completed; ratings re-assessed during peer review.	the written criteria allowed.	
Certainty of evidence	The record states that the certainty of findings would not be assessed.	GRADE assessment added (Section 2.8; Table 6).	Added in response to peer review.	Certainty rated very low.

Supplementary Table S8. Numerically reported estimates for alternative heatwave definitions and age groups

All dementia-specific mortality estimates for heatwave or extreme-heat exposure that are reported as numbers in the original publications are listed, to allow within-study comparison across thresholds, durations, and age groups. Estimates are odds ratios (95% CI) unless stated. The estimate selected by the hierarchy is shown in bold. These estimates are derived from the same deaths within each study and are not independent.

Definition	Estimates (95% CI)	Location in the original publication
**Gao et al. (2024) [52]; cumulative lag 0–13 d; no duration criterion**		
Hot-day excess (HDE), overall	P90: 1.79 (1.57–2.04); P92.5: 1.72 (1.52–1.96); **P95: 1.62 (1.43–1.83)**; P97.5: 1.46 (1.27–1.68)	Table 1
Hot-night excess (HNE), overall	P90: 1.56 (1.39–1.74); P92.5: 1.52 (1.36–1.70); P95: 1.46 (1.30–1.63); P97.5: 1.38 (1.22–1.55)	Table 1
HDE (P95) by age	<65 y: 1.09 (0.67–1.77); 65–74 y: 1.61 (1.13–2.28); ≥75 y: 1.66 (1.44–1.91)	Table 2
HDE (P95) adjusted for $PM_{2.5}$ and ozone	1.59 (1.39–1.83)	Table 3
**Li et al. (2026) [51]; 92.5th percentile, ≥4 d; cumulative lag 0–14 d**		
By outcome	**Dementia: 1.82 (1.61–2.06)**; Alzheimer's disease: 1.97 (1.59–2.44); non-Alzheimer dementias: 1.75 (1.50–2.03); for comparison, total neurodegenerative disease: 1.77 (1.58–1.98)	Results text (Section 3.2)
Other definitions and age strata	Shown graphically only	Supplementary Figures S4–S8 of the article
**Liu et al. (2024) [53]; cumulative lag 0–10 d**		
≥2 d	P90: 1.54 (1.43–1.66); P92.5: 1.58 (1.46–1.70); P95: 1.74 (1.60–1.88); P97.5: 2.17 (1.95–2.40)	Table 2
≥3 d	P90: 1.53 (1.42–1.65); P92.5: 1.57 (1.46–1.69); **P95: 1.75 (1.61–1.90)**; P97.5: 2.23 (2.00–2.49)	Table 2
≥4 d	P90: 1.49 (1.39–1.60); P92.5: 1.57 (1.46–1.69); P95: 1.79 (1.64–1.94); P97.5: 2.33 (2.08–2.62)	Table 2
P95, ≥3 d, added effect (further adjusted for daily mean temperature)	1.35 (1.19–1.54)	Table 2
P95, ≥3 d, by age	<85 y: 1.73 (1.54–1.95); ≥85 y: 1.77 (1.58–1.99)	Table 4
**Tao et al. (2025) [54]; same day**		
P90, ≥2 d	**Added effect: 1.13 (0.95–1.34)**; intensity-related effect: 1.51 (0.94–2.43). For comparison, all mental disorders: added effect 1.03 (0.91–1.11, as printed), intensity-related effect 1.59 (1.21–2.09).	Results text
P95, ≥3 d; P99, ≥4 d	Dementia-specific estimates shown graphically only	Figure 2C
Additional report [68] (2016–2019; F00–F09; heatwaves defined by a health-based excess heat factor; OR at the 90th percentile of the exposure–response curve; same day)	Daytime heatwave: 1.13 (0.66–1.93); nighttime heatwave: 0.61 (0.20–1.87); compound heatwave: 1.10 (0.69–1.77)	Table S2 of [68]
**Xu et al. (2019) [55]**		
P95, ≥2 d, post-discharge death	**3.69 (1.76–7.65)**, reported as an increase of 269% (76–665%)	Abstract
P90 and P97, ≥2 d; lags 0–7 d	Shown graphically only	Figure 2
**Zhang et al. (2024) [49]; cumulative odds ratio, lag 0–7 d; all aged ≥60 y**		
≥2 d	P90: 1.140 (1.118–1.163); P95: 1.250 (1.220–1.281); P97.5: 1.412 (1.366–1.460)	Figure 2a–c
≥3 d	P90: 1.143 (1.120–1.166); **P95: 1.268 (1.236–1.300)**; P97.5: 1.437 (1.386–1.489)	Figure 2a–c
≥4 d	P90: 1.145 (1.122–1.168); P95: 1.287 (1.253–1.322); P97.5: 1.459 (1.403–1.518)	Figure 2a–c

Definition	Estimates (95% CI)	Location in the original publication
P95, ≥3 d, by age	60–74 y: 1.286 (1.205–1.372); ≥75 y: 1.265 (1.230–1.300)	Figure 2b

Abbreviations: CI, confidence interval; d, consecutive days; P90–P99, percentiles of the local temperature distribution; $PM_{2.5}$, particulate matter with an aerodynamic diameter ≤2.5 μm; y, years.

Supplementary Figure S1. Funnel plot of the six study-level estimates

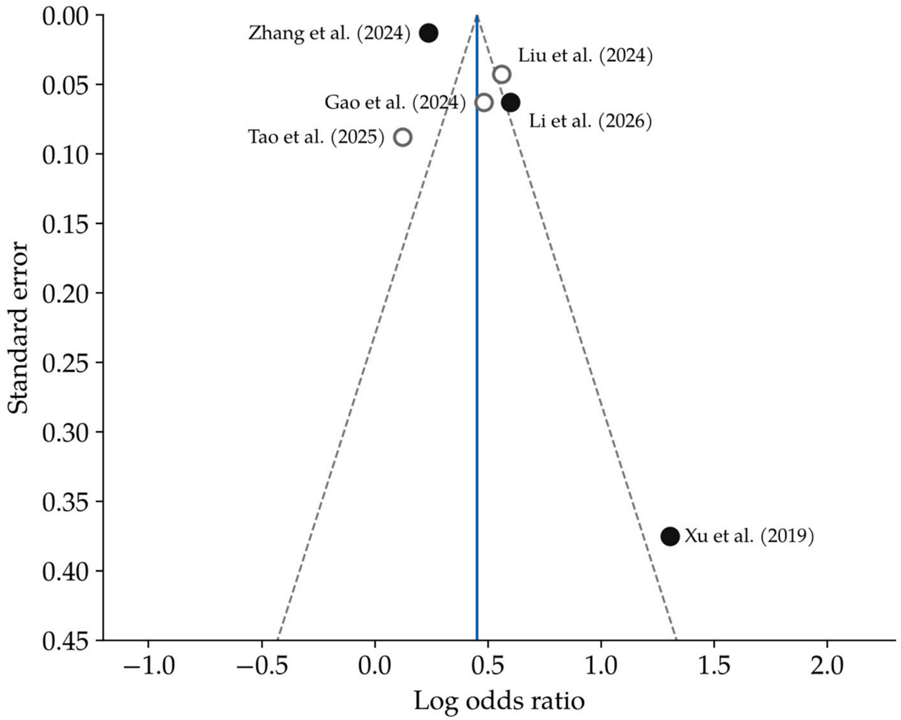

Each point shows one study: the horizontal axis is the natural logarithm of the study-level odds ratio, and the vertical axis is its standard error (inverted, so that larger and more precise studies appear toward the top). Filled circles are the three studies pooled in the primary analysis, and open circles are the eligible studies used only in sensitivity analyses. The vertical line marks the random-effects pooled estimate of all six studies (restricted maximum-likelihood estimation; odds ratio 1.57), and the dashed lines mark the region within which 95% of studies would be expected to lie in the absence of heterogeneity and small-study effects. The smallest study (Xu et al.) lies to the right of the pooled estimate, producing visual asymmetry. With only six studies and high heterogeneity ($I^2 > 90\%$), this asymmetry cannot be distinguished from genuine between-study differences, and no inference about publication bias was drawn from the plot. Studies shown: Zhang et al. (2024) [49], Li et al. (2026) [51], Gao et al. (2024) [52], Liu et al. (2024) [53], Tao et al. (2025) [54], and Xu et al. (2019) [55].

Note on the funnel plot in the initial submission: that plot, based on six studies, was exported from Comprehensive Meta-Analysis with the trim-and-fill option enabled; the three filled circles on the left were imputed studies and the lower diamond was the adjusted estimate. The procedure had not been planned or described. Reproduced in R on the initially selected estimates of the five studies of the initial submission that remain eligible (File S1), trimming under a fixed-effect model imputed two studies (log odds ratios −0.28 and −1.03) and gave adjusted pooled estimates of 1.15 (95% CI 1.13–1.17) under a fixed-effect model and 1.21 (95% CI 0.95–1.55) under a random-effects model. Trim-and-fill is unreliable with fewer than 10 studies and substantial heterogeneity; the values are reported for transparency only.
